# Nutraceutical Study on *Maianthemum atropurpureum*, a Wild Medicinal Food Plant in Northwest Yunnan, China

**DOI:** 10.3389/fphar.2021.710487

**Published:** 2021-07-30

**Authors:** Li Xu, Yizhou Wang, Yuanyuan Ji, Ping Li, Wujisiguleng Cao, Shibiao Wu, Edward Kennelly, Chunlin Long

**Affiliations:** ^1^ Key Laboratory of Ecology and Environment in Minority Areas, National Ethnic Affairs Commission, Minzu University of China, Beijing, China; ^2^ College of Life and Environmental Sciences, Minzu University of China, Beijing, China; ^3^ Key Laboratory of Ethnomedicine, Ministry of Education, Minzu University of China, Beijing, China; ^4^ Department of Biological Sciences, Lehman College City University of New York, New York, NY, United States; ^5^ Ph.D. Programs in Biochemistry, Biology, and Chemistry, The Graduate Center, City University of New York, New York, NY, United States

**Keywords:** *Maianthemum atropurpureum*, ethnic people, east Himalayas, nutraceutical profile, steroid saponins

## Abstract

*Maianthemum atropurpureum* (Franch) LaFrankie (Asparagaceae), called *nibai* in Tibetan or *dongka* in Drung or *zhu-ye-cai* in local Chinese, is a wild vegetable consumed by the Tibetan people and other ethnic groups in Northwest Yunnan, China. It is also a traditional medicinal plant used by different linguistic groups for antimicrobial purposes. However the nutritional and phytochemical compositions of this important medicinal food plant have not been well studied previously. In this study, the nutrient content for *nibai* was determined by the China National Standards (GB) methods, and the phytochemical analysis involved multiple chromatographic and spectral methods including LC-TOF-MS analysis. Dried *nibai* is a rich source of protein (ca. 24.6%), with 18 of the 21 common amino acids. The amino acid content of *nibai* can reach up to 17.9/100 g, with the essential amino acids as major contributors, corresponding to 42.3% of the total amino acids. *Nibai* contains rich mineral elements, dietary fiber, vitamins, β-carotene, carbohydrates, and lipids. The phytochemical content of *nibai* was examined by conventional isolation strategies, as well as HR-ESI-TOF-MS to detect and identify 16 compounds including nine steroid saponins and seven flavonoids. Among these compounds, uridine, adenosine, guanosine, and β-methyl-6-methyl-d-glucopyranoside were found from the genus *Maianthemum* for the first time. These results help to demonstrate that the local people’s practice of consuming *Maianthemum atropurpureum* is reasonable due to its high levels of vitamins, minerals, essential amino-acids, and phytochemicals. *Nibai* may be further developed in Tibet and surrounding regions, and beyond as a health food, nutraceutical, and/or dietary supplement product.

## Introduction

Wild edible plants play an important role in furthering food security and improving the nutrition in the diets of people around the world, especially in poor rural communities (Lulekal et al., 2011). The utilization of wild edible plants is receiving more and more attention, not only due to their health benefits but also the opportunities they may present to rural economies (Multari et al., 2016). In daily life, wild vegetables may be a significant source of nutrients, including minerals, vitamins, fiber, and essential amino acids which are critical for good health, but often their nutrient and phytochemical content are not well studied (Turan et al., 2003). Many edible plants are also considered medicinal herbs (Liu et al., 2018; Luo et al., 2019; Sarker and Oba, 2019a, Sarker and Oba, 2020a; Sarker and Oba, 2020b). Some wild vegetables are gaining more widespread popularity due to their unique flavors, colors, and potential health properties (Costa et al., 2013; Sarker and Oba, 2019b).

The ethnic groups including Nu, Dulong (Drung), Lisu, Yi, Pumi, Bai, Naxi and Tibetan as well in northwest Yunnan (including Nujiang, Diqing, Dali and Lijiang provinces) of China, reside at high elevation areas with mountains and deep valleys. To better survive in these extreme conditions, local ethnic people often turn to wild (uncultivated) plants to supplement their diet and therebey enrich their food diversity. Ahmad and Pieroni found that certain wild edible plants have been embraced by a particular local culture because of their traditionally acquired knowledge-based principles, feelings, and manners (Ahmad and Pieroni, 2016). Moreover, wild foods contribute to overcoming periods of famine, and dishes made of wild plants can be very healthy by providing local people with various essential nutritious elements, such as vitamins and minerals.

**FIGURE 1:**
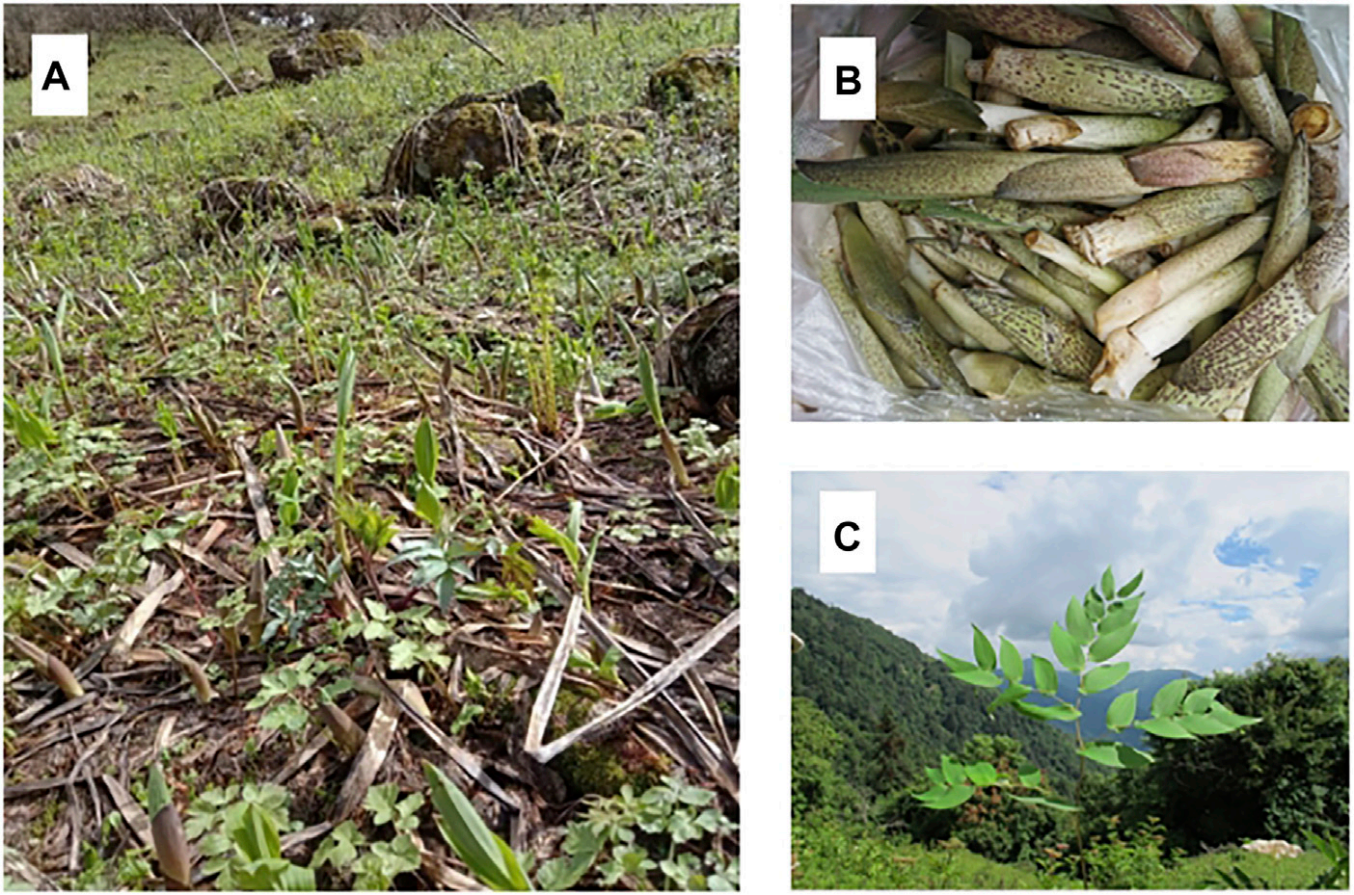
The plant of *Maianthemum atropurpureum*. **(A)** Wild habitat; **(B)** Shoots sold on market; **(C)** Mature leaves (photographed by the authors).

The local people in northwest Yunnan collect the young shoots (tender aerial parts) of a group of seven species in *Maianthemum* Web. for food, including *M. atropurpureum* (Franch.) LaFrankie, *M. purpurea* (Wall.) LaFrankie, *M. oleracea* (Baker) LaFrankie, *M. tatsienense* (Franch.) LaFrankie, *M. forrestii* (W.W. Sm.) LaFrankie*, M. fuscum* (Wall.) LaFrankie*,* and *M. henryi* (Baker) LaFrankie (Ju et al., 2012), belonging to the family Asparagaceae. Among them, *M. atropurpureum* is the most well-known, and considered to taste the best. *Maianthemum atropurpureum* (Franch.) LaFrankie, called *nibai* in Tibetan, *dongka* in Drung, *zhu-ye-cai* in local language, or *gao-da-lu-yao* in Mandarin, a flavorful seasonal wild vegetable with unique flavors. It grows at high attitude on mountains, and can be collected in May when the snow has just melted (Figure 1). It was one of the major vegetables consumed by local herdsmen in the Tea Horse Road trade route. The previous studies reported that everyday about 2 tons (2000 kg) of would be sold in a single market of a town in northwest Yunnan, while more *Maianthemum atropurpureum* (*nibai*) were collected and directly consumed by the local people (Gui et al., 2000a; Gui et al., 2000b). Now it is considered by Tibetan, Nu, Drung, Lisu, Bai, Yi, Pumi, and Naxi people as a delicacy used to celebrate festivals and entertain guests. There are many ways to prepare *M. atropurpureum*: the fresh shoots can be used in soups, stir-fried with pork, or eaten raw as salad, and the dried shoots can be served in wintertime hot pot dishes.

*Maianthemum atropurpureum* is also considered to be an excellent medicine for annealing, heat-clearing, detoxicating, and lowering blood pressure in traditional Chinese medicine practice. The rhizome, as a folk medicine, has been used to treat lung ailment, rheumatism, menstrual disturbance, mammitis, cuts, bruises, kidney diseases, and also to activate blood circulation and to alleviate pain (Jiang, 1977; State Administration of Traditional Chinese Medicine, 1999). The minerals, vitamins, essential amino-acids, carbohydrates and lipids in leaves of *Maianthemum atropurpureum* (*nibai*) have been preliminarily investigated (Gui et al., 2000a). In addition, steroid saponins, nucleosides and flavonoids, were isolated from *nibai* (Yang, 2003; Zhang et al., 2006; Yang et al., 2009).

Seven new steroidal saponins, atropurosides A–G, with new polyhydroxylated aglycones, were identified from the rhizomes of *Maianthemum atropurpureum*, together with a known saponin and dioscin. Among them, atropurosides B and F showed moderate inhibitory activity against *Candida albicans*, *Candida glabrata*, *Cryptococcus neoformans*, and *Aspergillus fumigatus*, while dioscin was more active against *C. albicans* and *C. glabrata* (Zhang et al., 2006). These three compounds are moderately cytotoxic. It appears that the antifungal activity of these steroidal saponins correlates with their cytotoxicity against mammalian cells (Zhang et al., 2006).

Thesecondary metabolites from young edible shoots of *Maianthemum atropurpureum* have not been reported. Further work on *Maianthemum atropurpureum*, therefore, is necessary since its bioactive constituents are not fully understood. Its nutritional composition also need to be re-examined based on modern standard techniques. This study investigated the nutritional content and chemical constituents of this typical medicinal and food plant valued by the Tibetan, Nu, Drung and other linguistic groups, and discuss its potential to be developed as new functional food or nutraceutical.

## Materials and Methods

### Samples and Sample Preparation

The aerial parts of *Maianthemum atropurpureum* (Franch.) LaFrankie were collected in Gongshan County, Nujiang Prefecture, NW Yunnan, China, in May 2013 and identified by Prof. Chun-Lin Long (Minzu University of China). The voucher specimen (No. 20130584) was deposited at the Herbarium of Minzu University of China. Some of the sample (300 g) was boiled with salt water and dried for the determination of nutrient content. The remaining sample (8.5 kg) was dried in the Sun directly for phytochemical studies.

### Solvents, Reagents and Standards

The general laboratory solvents, including methanol (MeOH), ethanol (EtOH), petroleum ether (Pet), ethyl acetate (EtOAc), n-butyl alcohol (n-BuOH), acetone, chloroform (CHCl_3_), and dichloromethane (DCM) were of analytical grade and were purchased from Beijing Chemical Works (Beijing, China). Chromatography and extraction solvents (acetonitrile, methanol, formic acid) were of HPLC grade obtained from VWR Inc (Bridgeport, PA, United States). UPLC grade water was prepared by using the Millipore Milli-RO 12 system (Millipore, Bedford, United States). All pure isolated compounds were characterized by HR-ESI-MS, ^1^H NMR, and ^13^C NMR spectroscopic methods for identification purposes.

### Determination of Proximate Composition

Percentages of moisture was determined by vacuum oven under 105°C for 2 h to constant weight per the China national standards (GB) method (GB 5009.3-2010) (Zhang et al., 2009). Ash was determined by direct analysis according to the China standards method (GB 5009.4-2010), the sample was fully carbonized with a low flame until no smoke was produced, and weighed. The dry sample was then placed in a muffle furnace to burn under 550 ± 25°C for 4 h, removed, and placed into a desiccator for 30 min after cooling down to 200°C. The whole process was repeated until to a constant weight (Nie et al., 2014). Total lipid content was determined by Soxhlet extraction using the China standards method (GB/T 5009.6-2003) (Wang et al., 2005; Liu et al., 2015). Protein content was determined by Kjeldahl nitrogen using the China standards method (GB/T5009.5-2010) (Zhang, 2008), and the percentage of crude protein was estimated as the total nitrogen content multiplied by the conversion factor 6.25. Total carbohydrates were calculated by subtracting the total percentage of other components (saccharides, starch and dietary fiber) from 100.

### Determination of Dietary Fiber

Total fiber, soluble fiber, and insoluble fiber contents were determined by Dietary Fiber Determination System (VELP GDE + CSF6, Italian) using the GB/T 5009.88-2008 method (Zhou, 2014).

### Determination of Minerals, Vitamins and *β*-carotene

Minerals, vitamins, and *β*-carotene were analyzed according to corresponding China standard methods GB/T (Wang et al., 2005), among which K, Na, Fe, Mn, Mg, Ca, Cu and Zn were determined using inductively coupled plasma-mass spectrometery (ICP-MS) (Wang et al., 2005); P and vitamin C were determined by ultra-violet and visible spectrophotometery (UVPC) (Wang et al., 2005); Cr was determined using an atomic absorption spectrometer (AAS) (Wang et al., 2005); vitamin A was determined using ultra performance liquid chromatography (UPLC) (Wang et al., 2005); vitamin B_2_ was determined using molecular fluorescence photometer (Wang et al., 2005). The determination of *β*-carotene was carried out by high-performance liquid chromatography (HPLC) as previously reported (Sun et al., 2009).

### Determination of Amino Acids

Amino acids were measured according to the method GB/T 5009.124-2003 by Amino acid Analyzer (Hitachi L-8900, Japan) (Wang et al., 2005; Xing et al., 2012). Data were expressed as milligrams of amino acid per 100 g of *nibai*.

### Isolation and Identification of Chemical Compounds

Dried and powdered samples of *Maianthemum atropurpureum* (*nibai*) (8.5 kg) were extracted five times with 70% EtOH under reflux to give an ethanol extract. After evaporation under reduced pressure, the extracted residue was suspended in water and partitioned successively with petroleum ether, ethyl acetate, and n-BuOH. The n-BuOH extract (109 g) was subjected to silica gel (200–300 mesh) column chromatography eluting with EtOAc-acetone gradient (1:0→0:1) and pooled based on TLC profiles to yield seven fractions (Fr.1-Fr.7). Fr.2 (3.24 g) was dissolved with MeOH, and the insoluble part was recrystallized with MeOH and gave compound 1. The soluble part was subjected to silica gel (200–300 mesh) column chromatography eluting with CH_2_Cl_2_-MeOH gradient (25:1→2:1) to yield Fr.2.3 and Fr.2.5. Fr.2.3 was purified by Sephadex LH-20 chromatography using MeOH as the eluent to yield compound 3. In addition, Fr.2.5 was repeated chromatographed by silica gel column under different gradient conditions with CH_2_Cl_2_-MeOH to give compounds 4, 5, and 7. The insoluble part in Fr.3 (3.01 g) was also recrystallized with MeOH and gave compound 2, and the soluble part was further purified by successive silica gel column chromatography with CH_2_Cl_2_-MeOH (20:1, 15:1, 10:1) followed by CH_2_Cl_2_-MeOH (15:1, 10:1), yielding 8 mg of compound 6. Fr.5 (4.47 g) was subjected to Si gel column chromatography with CH_2_Cl_2_-MeOH (18:1, 15:1, 10:1) and pooled based on TLC profiles to yield five fractions (Fr.5.1-Fr.5.5). Fr.5.3 was purified by silica gel column chromatography (15:1, 12:1) to yield compound 10. Fr.5.4 was repeated silica gel medium pressure liquid chromatography gradient eluted with a MeOH-H_2_O solvent system (5, 10, 20, 40, 60, and 100%), followed by preparative HPLC with 50% MeOH (MeOH-H_2_O) and further purified by silica gel column to yield compounds 8 and 9.

### Liquid Chromatography Time-of-Flight Mass Spectrometry Analysis

LC-TOF-MS analysis of the n-BuOH extract was performed using a Waters (Milford, MA, United States) Alliance 2695 system equipped with 2695 separation module units and 2998 PDA detectors using a 100 mm × 2.0 mm, 2.5 µm Phenomenex Synergi Hydro-RP 100 A column with 3 mm × 4.0 mm Phenomenex Security Guard column (Torrance, CA United States). The mobile phase consisted of solvents (A) 0.1% aqueous formic acid solution and (B) acetonitrile using a stepwise gradient elution of 3% B for 5 min, 3%–15% B at 5–15 min, 15% B for 5 min, 15%–45% B at 21–35 min, 45%–90% B at 35–40 min, and this proportion of solvent kept for 10 min. The UV-Vis spectra were recorded from 190 to 700 nm. The flow rate and the injection volume were 0.2 ml/min and 10 μL, respectively. Both column and sample temperatures were 25°C. LC/MS-grade methanol was used to redissolve the freeze-dried sample, and then brought up to 2 mg/ml and filtered using 25 mm syringe filter (0.45 µm PTFE membrane) prior to injection.

High-resolution electrospray ionization time of flight mass spectrometry (HR-ESI-TOF-MS) was performed using an LCT Premier XE TOF mass spectrometer (Waters, Milford, MA) equipped with an ESI interface and controlled by Mass Lynx V4.1 software. Mass spectra were acquired in both positive and negative modes over range m/z 100–1500. The capillary voltages were set at 3000 V (positive mode) and 2700 V (negative mode), respectively, and the cone voltage was 30 V. Nitrogen gas was used for both the nebulizer and desolvation. The desolvation and cone gas flow rates were 600 and 20 L/h, respectively. The desolvation temperature was 400°C, and the source temperature was 120°C.

## Results and Discussions

### Nutrient Composition and Dietary Fiber

The nutrient composition and dietary fiber content of *Maianthemum atropurpureum* (Franch.) LaFrankie (*nibai*) are listed in Table 1. Insoluble dietary fiber was the predominant component (26.0/100 g), followed by protein (24.6/100 g), while carbohydrate and lipid contents were much lower. The dietary fiber content is comparable with that of fiddlehead fern (*Pteridium aquilinum* var. *latiusculum*) with 25.5/100 g, one of the most important wild food plants in China (Liu et al., 2012), and higher than that of spinach (*Spinacia oleracea*) with 12.7/100 g. The protein and carbohydrate content among three species are very different; *nibai* has the highest protein content (24.6/100 g), and the lowest carbohydrate content (23.8/100 g). The protein and carbohydrate content of *nibai* is much higher than different vegetable amaranth species, such as *Amaranthus blitum* (Sarker and Oba, 2020c), stem amaranth (Sarker et al., 2020a), green morph amaranth (Sarker et al., 2020b) and red morph amaranth (Sarker and Oba, 2019c).

**TABLE 1:** Compositional and nutritional characteristics of *zhu-ye-cai* compared with other well-known edible greens. (Values per 100 g of *zhu-ye-cai* dried after boiling with salted water)

Parameters	Unit	*zhu-ye-cai*	fiddlehead^a^	spinach^a^
Approximate composition				
Moisture	G	7.22	nd	Nd
Ash	G	13.5	nd	Nd
Crude lipid	G	4.9	0.9	0.6
Crude protein	G	24.6	6.6	6.4
Total carbohydrate	G	23.8	54.2	63
Dietary fiber				
Total dietary fiber	G	26.1	25.5	12.7
Soluble dietary fiber (SDF)	G	0.07	nd	Nd
Insoluble dietary fiber (IDF)	G	26.0	nd	Nd
Minerals				
K	Mg	1800	59	919
Na	Mg	2490	20.4	242
Ca	Mg	252	851	411
Mg	Mg	231	82	183
Fe	Mg	20.3	23.7	25.9
Mn	Mg	5.98	2.31	1.61
Zn	Mg	4	18.11	3.91
Cu	Mg	6.62	2.79	2.08
Cr	Mg	0.84	nd	Nd
P	Mg	313	253	222
Vitamins and β- carotene				
β-carotene	Mg	7.95	nd	Nd
Vitamin A	Mg	9.8	0	598
Vitamin B_2_	Mg	0.2	nd	Nd
Vitamin C	Mg	2	3	82

a From *Chinese Food Composition Tabl*e.^35^.

All of these vegetables are dehydrated, and *zhu-ye-cai* is dried after boiling with salt water so the Sodium content is extremely high; nd means not detected (below the detection level).

Dietary fiber is indispensable to a healthy diet, and its health benefits have been known for decades (Le et al., 2016). In the gastrointestinal tract, dietary fibers can promote intestinal peristalsis to accelerate the removal of carcinogenic substances and toxic matter, and reduce bacterial growth. Dietary fiber can also help to reduce cholesterol levels, prevent obesity and chronic diseases (Patil et al., 2009). Dietary fiber significantly contributed to the cure of constipation, digestibility, and palatability (Sarker et al., 2020c; Sarker and Oba, 2020d; Sarker and Oba, 2020e). *Nibai* has a high content of protein, along with a low carbohydrate content, making it a potentially healthy food plant for people looking to reduce carbohydrate intake. With the growing obesity epidemic in developed nations, foods with high protein and diet fiber content, and low carbohydrate and lipid content has been advocated by certain nutritionists. Thus, *nibai* could potentially be considered a functional food which can meet the modern healthy diet requirement.

### Minerals, Vitamins and *β*-Carotene

Ten minerals have been detected in *nibai*, and five of them are essential microelements of human body, including Fe, Zn, Cu, Cr, and Mg (Table 1). The content of Fe (20.3 mg/100 g) is comparable with that of spinach (25.9 mg/100 g) and fiddlehead (23.7 mg/100 g) which are commonly considered Fe-rich vegetables. However, it is much higher than commonly considered Fe rich *Amaranthus hypochondriacus* (Sarker and Oba, 2020d). The Mg, Cu and P levels in *nibai* are the highest, when compared to spinach and fiddlehead fern. Mg, Cu and P were much higher than vegetable amaranth (Sarker and Oba, 2020e). Zn levels in *nibai* are similar to spinach, but both are significantly lower than fiddlehead. However, Zn content was much lower than vegetable amaranth (Sarker et al., 2020c). It has been estimated that half the world’s population is iron deficient (Karakoy et al., 2012). Therefore, *nibai* could be a very useful dietary source for Fe and other nutrients to help maintain good health for people with certain nutrient deficiencies. Vitamins serve many physiological functions and are of the essence to maintain health. Compared with spinach and vegetable amaranth, the contents of vitamins A, B_2_, and C in *nibai* are lower, but it is still an important source of vitamins for Tibetans and other linguistic groups through the famine and the food shortages caused by the extreme environment. Consuming *nibai* contribute to prevent vitamins and trace elements deficiencies and maintain the normal bodily functions.

### Amino Acids

The 18 amino acids identified and quantified in *nibai* are presented in Table 2, in which eight are essential to humans. Tryptophan was detected in *nibai* for the first time (Gui et al., 2000a; Gui et al., 2000b). The total amino acid content is up to 17.9 g/100 g, and the percentage of essential amino acids in total amino acids (E/T) reach up to 42.29%, which is higher than that of soybean (33%). According to the ideal model proposed by WHO/FAO, the ratio of E/T of good quality protein is about 40%, and the ratio of E/N (the percentage of essential amino acids to nonessential amino acid) is above 60% (Zhang et al., 2013). The composition of amino acid of *nibai* meets this ideal model. Additionally, the content of glutamic acid is the highest (2.98 g/100 g), followed by aspartic acid (1.73 g/100 g), leucine (1.71 g/100 g), lysine (1.32 g/100 g), and alanine (1.29 g/100 g), respectively.

**TABLE 2:** Amino acids of *zhu-ye-cai* (Values per 100 g of *zhu-ye-cai* dried after boiling with salted water).

Amino acids	unit	*zhu-ye-cai*
Aspartic acid (Asp)	g	1.73
Threonine (Thr)*	g	0.73
Serine (Ser)	g	0.74
Glutamate (Glu)	g	2.98
Proline (Pro)	g	0.64
Glycine (Gly)	g	0.9
Alanine (Ala)	g	1.29
Cystine (Crs)	g	0.26
Valine (Val)*	g	1.24
Methionine (Met)*	g	0.11
Isoleucine (Ile)*	g	1.09
Leucine (Leu)*	g	1.71
Tyrosine (Tyr)	g	0.41
Phenylalanine (Phe)*	g	1.16
Lysine (Lys)*	g	1.36
Histidine (His)	g	0.34
Tryptophan (Try)*	g	0.17
Arginine (Arg)	g	0.92
Essential amino acids(E)	g	7.57
Total amino acids(T)	g	17.9
E/T	%	42.29
Lys/T	%	7.59

**Note** * indicates essential amino acids.

Glutamate and arginine play important roles in regulating gene expression, cell signaling, antioxidative responses, and immunity (Wu, 2010). Our work showed that *nibai* is a good source of amino acids, which can provide indigenous people with nearly all the amino acids that humans require. Four amino acids in *nibai*, aspartic acid and glutamate contribute to its umami taste, while glycine and alanine contribute to its sweet taste (Bu et al., 2013). The total content of these four amino acids in *nibai* was 6.9 g/100 g, accounting for 38.6% of the total amino acids, these four amino acid contribute a characteristic taste to the flavor of *nibai*.

### Phytochemical Constituents

**FIGURE 2:**
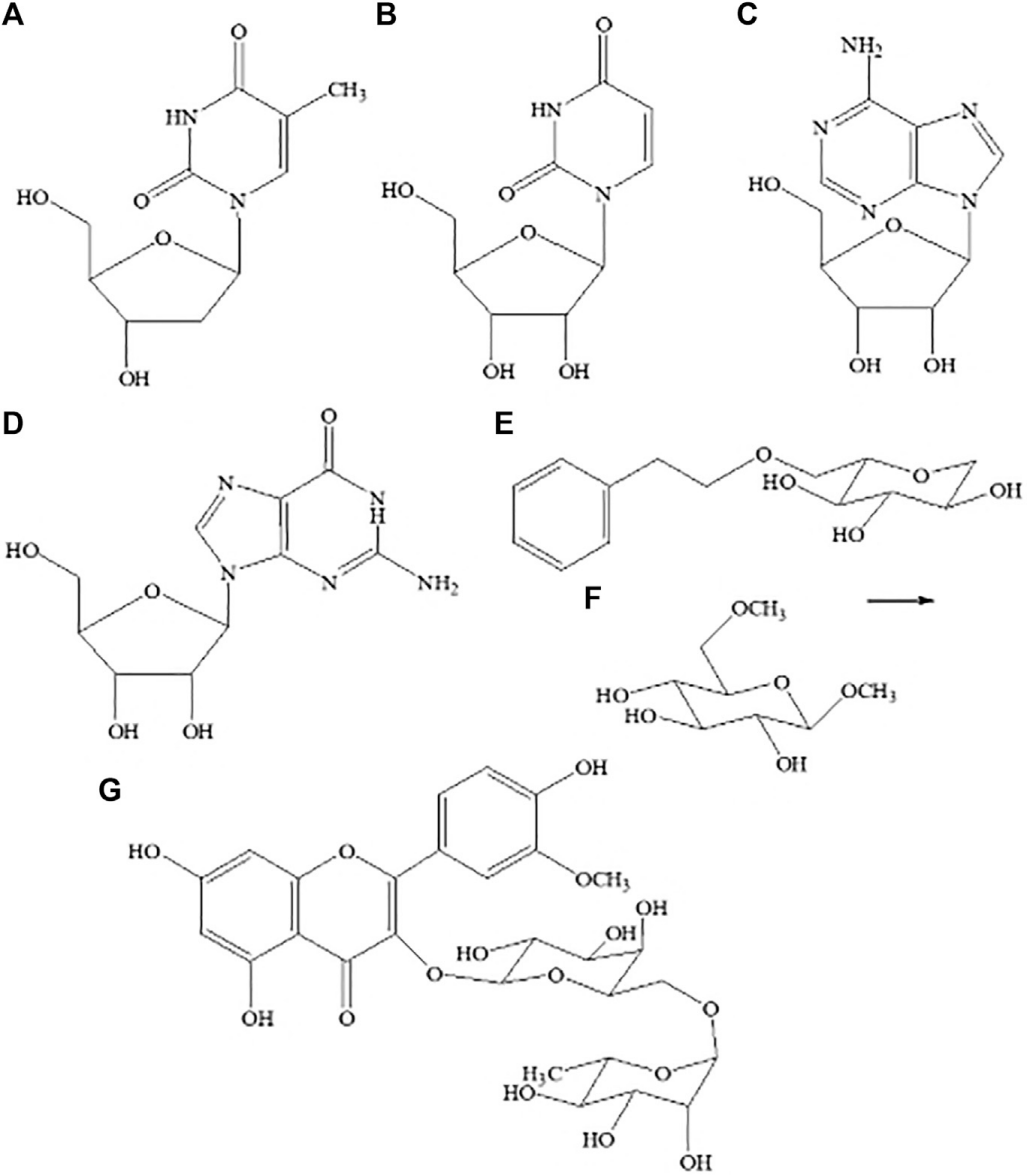
Structures of seven compounds isolated from *Maianthemum atropurpureum*. Thymidine **(A)**, uridine **(B)**, adenosine **(C)**, guanosine **(D)**, 2-Phenylethyl-*β*-D-glucopyranoside **(E)**, *β*-methyl-6-methyl-D-glucopyranoside **(F)**, isorhamnetin-3-*O*-robinobioside **(G)**.

Sixteen compounds were detected and tentatively identified using a method based on HPLC coupled with both PDA and HR-ESI-TOF-MS. The TIC chromatography of the n-BuOH extract of *M. atropurpureum* is displayed in the supplementary material (DataSheet 1). Their retention times, UV spectra, and exact mass spectral fragmental ions in positive and negative modes are shown in Table 3, and compared with literature values. Furthermore, the molecular formula was calculated based upon fragment ion peak data along with previous reports in SciFinder and other databases. The structures of part selected compounds are shown in Figure 2.

**TABLE 3:** LC-MS-TOF data of the compounds identified from the n-butonal extract of *Maianthemum atropurpureum*.

No	R.T. (min)	UV	[M + H]^+^ or [M-H]^-^ (M.F., ppm)	Adduct and fragmental ion exact masses [M-X]^+^ or [M-X]^-^ (M.F., ppm)	Identification	References
8	8.3	230, 256	611.1619 [M + H]^+^ (C_27_H_31_O_16_, 1.1)	633.1429 [M + Na]^+^ (C_27_H_30_O_16_ Na,-0.5); 465.1053 [M + H-146(Rha)]^+^ (C_21_H_21_O_12_, 4.3)	Rutin	Kaneta et al. (1980)
			609.1428 [M-H]^-^ (C_27_H_29_O_16_, -4.6)	655.1569 [M-H + HCOOH]^-^ (C_28_H_31_O_18_, 9.0)			
9	10.5	230, 256	465.1055 [M + H]^+^(C_21_H_21_O_12_, 4.7)	303.0497 [M + H- Glc]^+^ (C_15_H_11_O_7_,−2.6); 487.0862 [M + Na]^+^ (C_21_H_20_O_12_Na, 2.1)	Quercetin 3-O-galactoside	Kaneta et al. (1980)
			463.0916 [M-H]^-^ (C_21_H_19_O_12_, 8.4)	—			
10	13.1	230, 256	611.1620 [M + H]^+^(C_27_H_31_O_16_, 1.3)	633.1478 [M + Na]^+^(C_27_H_30_O_16_ Na,7.3); 465.1027 [M + H-146(Rha)]^+^ (C_21_H_21_O_12_, -1.3); 303.0521 [M + H-Rha-Glc]^+^(C_15_H_11_O_7_, -1.3)	Quercetin 3-O-glucoside 7-O-rhamnoside	Kaneta et al. (1980)
			609.1505 [M-H]^-^ (C_27_H_29_O_16_, 8.0)	655.1569 [M-H + HCOOH]^-^ (C_28_H_31_O_18_, 9.0)			
11	13.6	230, 256	625.1740 [M + H]^+^(C_28_H_33_O_16_,-4.6)	479.1211 [M-Rha + H]^+^ (C_22_H_23_O_12_,4.4); 317.0660 [M-Rha-Glc + H]^+^ (C_16_H_13_O_7_,-0.3); 647.1553 [M + Na]^+^(C_28_H_32_O_16_Na, -5.4)	Isorhamnetin-3-Rutinoside	El-Alfy et al. (1975)
			623.1763 [M-H]^-^(C_28_H_31_O_16_,- 0.3)	669.1641 [M-H + HCOOH]^-^ (C_29_H_33_O_18_,-3.9)			
12	14.6	—	755.4235 [M + H]^+^(C_39_H_63_O_14_,2.3)	593.3706 [M-Glc + H]^+^ (C_33_H_53_O_9_,2.7);	27-epi-Trikamsteroside A	Yokosuka et al. (2008)
13	15.0	—	—	871.4673 [M-H_2_O + H]^+^(C_44_H_71_O_17_, −2.1); 793.4024 [M-Xyl+2H_2_O + H]^+^ (C_39_H_69_O_16_,1.3); 725.4124 [M-Rha-H_2_O + H]^+^ (C_38_H_61_O_13_,1.7); 593.3668 [M- Xyl-Rha- H_2_O + H]^+^ (C_35_H_53_O_9_, −3.7); 413.3078 [M- Xyl-Rha-Gal- 2H_2_O + H]^+^(C_27_H_41_O_3_,5.3); 911.4673 [M + Na]^+^ (C_44_H_72_O_18_Na, 6.3)	(3β,5α,6β,25R)-spirostane-3,5,6-triol-3-O-β-D-apiofuranosyl-(1→ 3)-[α-l-rhamnopyranosyl-(1→2)]- β-D-glucopyranoside	Wu et al. (2012)
			887.4696 [M-H]^-^ (C_44_H_71_O_18_, 6.3)	933.4628 [M-H + HCOOH]^-^(C_45_H_73_O_20_,-7.2)			
14	15.9	—	901.4814 [M + H]^+^(C_45_H_73_O_18_,1.9)	923.4615 [M + Na]^+^ (C_45_H_72_O_18_ Na, −0.1); 739.4250 [M -Glc + H]^+^(C_39_H_63_O_13_, −2.6); 577.3737 [M-2Glc- + H]^-^(C_33_H_53_O_8_, −0.5)	Funkioside D	Yang et al. (2009)
		—	899.4673 [M-H]^-^ (C_45_H_71_O_18_,3.7)	945.4709 [M-H + HCOOH]^-^ (C_46_H_75_O_20_,1.5)	—	—
15	16.8	—	—	885.4833 [M + H-H_2_O]^+^(C_45_H_73_O_17_, −1.7); 739.4282 [M -Rha- H_2_O + H]^+^(C_39_H_63_O_13_, 1.8); 577.3737 [M-Gal-Rha- H_2_O + H]^+^(C_33_H_53_O_8_, −0.5); 415.3230 [M-Gal-Glc-Rha-H_2_O + H]^+^ (C_27_H_43_O_3_, 4.3); 925.4753 [M + Na]^+^ (C_45_H_74_O_18_Na, −2.2)	Slimacinoside C	Yang et al. (2009)
		—	901.4769 [M-H]^-^ (C_45_H_73_O_18_, −3.1)	947.4926 [M-H + HCOOH]^-^ (C_46_H_75_O_20_, 7.8)	—	—
16	17.7	230, 256	303.0494 [M + H]^+^(C_15_H_11_O_7_, -3.6)	—	Quercetin	Zhao et al. (2009)
		—	301.0342 [M-H]^-^ (C_15_H_9_O_7_, -2.0)	—	—	—
17	17.9	230, 256	287.0549 [M + H]^+^(C_15_H_11_O_6_, -2.4)	—	Luteolin	Zhao et al. (2009)
		—	285.0405 [M-H]^-^ (C_15_H_9_O_6_, 2.1)	—	—	—
18	18.1	—	—	739.4282 [M-Rha- H_2_O + H]^+^(C_39_H_63_O_13_, 0.0); 577.3745 [M-Gal-Rha-H_2_O + H]^+^ (C_33_H_53_O_8_,0.9); 415.3211 [M-Gal-Glc-Rha- H_2_O + H]^+^ (C_27_H_43_O_3_, −0.2); 397.3138 [M-Gal-Glc-Rha- H_2_O + H]^+^ (C_27_H_43_O_3_, −0.2); 925.4819 [M + Na]^+^ (C_45_H_74_O_18_Na, 5.0)	(3β,22α)-26-(β-D-glucopyranosyloxy)-22-hydroxyfurost-5-en-3-yl2-O-(6-deoxy-α-L-mannopyranosyl) -β-D-glucopyranoside
		—	901.4828 [M-H]^-^ (C_45_H_73_O_18_, 3.4)	947.4926 [M-H + HCOOH]^-^ (C_46_H_75_O_20_, 7.8)	—	—
19	18.7	230, 256	—	353.4832 [M + Na]^+^ (C_22_H_35_ O_2_ Na, 3.9)	1-Phenanthren-emethanol	Kitajima et al. (1982)
		—	329.4812M-H]^-^ (C_22_H_33_O_2_, −2.1)	—	—	—
20	18.9	230, 256	885.4838 [M + H]^+^(C_45_H_73_O_17_,-1.1)	907.4659 [M + Na]^+^ (C_45_H_72_O_17_Na, −0.9); 739.4293 [M-Rha + H]^+^(C_39_H_63_O_13_, 3.2); 577.3766 [M-Glc-Rha + H]^+^(C_33_H_53_O_8_, 4.5); 415.3199 [M-2Glc-Rha + H]^+^ (C_27_H_43_O_3_, −3.1)	Gracillin	Inoue et al., 1995; Zhao et al., 2011
		—	883.4691 [M-H]^-^ (C_45_H_71_O_17_, **-**4.0)	929.4808 [M-H + HCOOH]^-^ (C_46_H_73_O_19_, 6.7)	—	—
21	19.7	—	—	355 [M + Na]^+^ (C_22_H_37_ O_2_ Na, −3.4)	Ent-kaur-15-en-17-ol	Kitajima et al. (1982)
		—	331 [M-H]^-^ (C_22_H_35_O_2_,1.9)		—	—
22	19.9	230, 256	301.0717 [M + H]^+^ (C_16_H_13_O_6_, 1.7)	—	5,7,4′-trihydroxy-3′-methoxyflavone	Zhao et al. (2009)
		—	299.0573 [M-H]^-^ (C_16_H_11_O_6_, 5.7)	345.0599 [M-H + HCOOH]^-^ (C_17_H_13_O_8_, −3.2)	—	—
23	20.9	—	—	577.3740 [M-H_2_O + H]^+^(C_33_H_63_O_8_, 6.8); 617.3685 [M + Na]^+^ (C_33_H_54_O_9_Na,3.1)	26-O-β-D-glucopyranosyl-(25R)-furost-5-ene-3β, 22ξ, 26-triol	Wu et al., 2012
		—	593.3697 [M-H]^-^ (C_33_H_53_O_9_, 1.2)	639.3683 [M-H + HCOOH]^-^ (C_34_H_55_O_11_, −9.5)	—	—

Seven known compounds (Figures 2A–G) have been isolated using conventional column chromatography and elucidated on the basis of detailed spectroscopic analyses from *nibai*, including thymidine 1) (Yang and Liu, 2003), uridine 2) (Gong and Ding, 2005), adenosine 3) (Yang and Liu, 2003), guanosine 4) (Gong and Ding, 2005), 2-phenylethyl- *β*-D-glucopyranoside 5) (Kim et al., 2008), *β*-methyl-6-methyl-d-glucopyranoside 6) (Gong and Ding, 2005), and isorhamnetin-3-*O*-robinobioside 7) (Wang et al., 2010). Among them, compounds 2, 3, 4, 6 were found from the genus *Maianthemum* for the first time.

More compounds had been identified from *Maianthemum atropurpureum* comparing with current literatures (Yang and Liu, 2003; Gong and Ding, 2005; Kim et al., 2008). In particular, four compounds including uridine, adenosine, guanosine, and β-methyl-6-methyl-d-glucopyranoside had not been reported from *Maianthemum* in any previous references. Our methods adopted for isolating chemical compounds may be more appropriate.

Thymidine 1) was isolated as colorless acicular crystal. All NMR data are in agreement with literature data (Yang and Liu, 2003). The molecular formula was obtained *via* ESI-MS and deduced as C_10_H_14_N_2_O_5_ (observed m/z at 243 [M + H]^+^ (calcd for C_10_H_14_N_2_O_5_, 243). The identification of uridine 2) which was isolated as colorless acicular crystal was confirmed by comparing the ^13^C NMR data with literature and database values (Gong and Ding, 2005). ESI-MS m/z 245 [M + H]^+^, C_9_H_12_N_2_O_6._ Compared with that of 1, upfield shifts in the C-5 and C-2′ indicated that there was no methyl substitute group, but with ribose connected. Based on these data, compound 2 was identified as uridine. Adenosine 3) was white powder; ^1^H NMR and ^13^C NMR were in agreement with literature values. ESI-MS m/z 267 [M + H]^+^ (calcd for C_10_H_13_N_5_O_4_, 267) corresponded with those reported in the literature (Yang and Liu, 2003). Guanosine 4) was identified as white powder; ^1^H NMR and ^13^C NMR were in agreement with the literature (Gong and Ding, 2005); ESI-MS m/z 283 [M + H]^+^ (calcd for C_10_H_13_N_5_O_5_, 283) and was identical to literature data (Gong and Ding, 2005). 2-Phenylethyl-*β*-d-glucopyranoside 5) was gummy, colorless, solid; ^1^H NMR^13^C-NMR are in agreement with published values. ESI-MS m/z 285 [M + H]^+^ (calcd for C_14_H_20_O_6_, 285) was identical to literature data (Kim et al., 2008). *β*-methyl-6-methyl-d-glucopyranoside 6) was white powder; ^13^C NMR (75 MHz, MeOD): δ 103.5 (C-1), 78.2 (C-3), 74.1 (C-5), 73.7 (C-2), 72.5 (C-6), 71.9 (C-4), 66.5 (C6-OCH_3_), 64.5(C1-OCH_3_); ESI-MS m/z 208 [M] (calcd for C_8_H_16_O_6_). Comparing with NMR database, the ^13^C NMR data showed 75% similarity to that of ethyl-β-D-fructopyranoside, from which the hydroxyl substituent fructose was confirmed. The ^1^H NMR spectrum of 6 showed the presence of six hydrogens at δ1.74 and 2 carbons at δ 66.5 and 64.5, pointing to the existence of two methoxy groups. Comparing all the carbon chemical shift data of *β*-methyl-d-glucopyranoside with 6-*O*-methyl-*β*-d-glucopyranoside, the data were in agreement with those obtained from the literature data (Gong and Ding, 2005). Therefore, 6 was identified as *β*-methyl-6-methyl-d-glucopyranoside. Isorhamnetin-3-*O*-robinobioside (7): pale yellow crystal; ^1^H NMR and ^13^C NMR are in agreement with published data (Kaneta et al., 1980), ESI-MS m/z 467 [M + Na]^+^ (calcd for C_28_H_32_O_16_, 467) (Wang et al., 2010).

### Biological Activities

The biological activities of *nibai* are attributed to its diverse bioactive components, especially the main constituents. According to this study and previous literature, the primary chemical components of *M. atropurpureum* are steroid saponins, nucleosides, and flavonoids, especially the large polarity constituents, some of which have considerable biological activities. For instance, the smilacinoside A, aspidistrin, and funkioside D isolated from the aerial parts of *M. atropurpureum* exhibited prominent activity *in vitro* cytotoxicity against K562 tumor cell line with IC_50_ values of 1.09, 0.47, and 2.93 μg/ml, respectively (Yang et al., 2009). Nucleosides also exerted a variety of biological activities, such as antitumor (Ogawa et al., 1998), antivirus (Ruth and Cheng, 1981) and gene therapy effects (Parker et al., 1997). Thymidine, for instance, is the nucleus of stavudine and zidovudine which are both anti-AIDS drugs (Ross et al., 2001). Moreover, adenosine has a range of significant pharmacological effects, including making blood vessels diastolic, lowering blood pressure, slowing heart rate, inhibiting platelet gathering, relaxing vascular smooth muscle, improving cardio-cerebral blood circulation, preventing arrhythmia, inhibiting the release of neurotransmitters, and adjusting the activities of adenosine activation enzyme (Layland et al., 2014). Flavonoids, such as rutin, quercetin, luteolin, and their glycosides are the most common and widely distributed group of plant phenolics (Sarker and Oba, 2018a; Sarker and Oba, 2018b). Many studies have demonstrated their antioxidant, scavenging free radical, anti-inflammatory, antibacterial, antiviral, and immunomodulatory effects (Sarker and Oba, 2020f; Sarker and Oba, 2020g; Sarker and Oba, 2021). All of these bioactive constituents as well as vitamin C, β-carotene and several minerals (Fe, Zn, and Mn), along with amino acids have contributed to *nibai’*s medicinal effects and thus demonstrate the current utilization of this wild plant to treat diseases and enhance immunity in humans.

Many studies have correlated diet and certain chronic diseases such as cancer, cardiovascular disease, diabetes, and osteoporosis. Bioactive compounds from edible plants have the potential to prevent certain chronic conditions (Patil et al., 2009; Sarker and Oba, 2018c; Sarker and Oba, 2018d; Sarker and Oba, 2018e). *Nibai*, *dongka* or *zhu-ye-cai* (*M. atropurpureum*), an endemic species to the Hengduan Mountains, is a less common wild vegetable rich in nutrient and phytochemical content. It plays an important role not only in providing local Tibetans and other linguistic groups with various essential nutrition elements, but may also contribute to maintaining health, promoting immunity, and preventing several kinds of diseases. Consequently, *nibai* can be considered a promising new functional health food and/or nutraceutical.

## Data Availability

The datasets presented in this article are not readily available because, All datasets had been included in the paper. Requests to access the datasets should be directed to long@mail.kib.ac.cn or long.chunlin@muc.edu.cn.

## Abbreviations

GB, national standard method of the people’s republic of China (initial of Guo Biao); MS/MS, mass spectrometry/mass spectrometry; NMR, nuclear magnetic resonance; PDA, photo-diode array; HR-ESI-TOF-MS, high resolution electrospray ionization time-of-flight mass spectrometry; TIC, total ion currency chromatograms; ESI-MS, electrospray ionization mass spectrometry; LC-TOF-MS, liquid chromatography time-of-flight mass spectrometry; MFCs, minimum fungicidal concentrations.
