# Supplementary figures and images for: Nutraceutical Study on Maianthemum atropurpureum, a Wild Medicinal Food Plant in Northwest Yunnan, China
Source: Front Pharmacol. 2021 Jul 30;12:710487. doi: 10.3389/fphar.2021.710487 (PMC8363226; doi:10.3389/fphar.2021.710487)

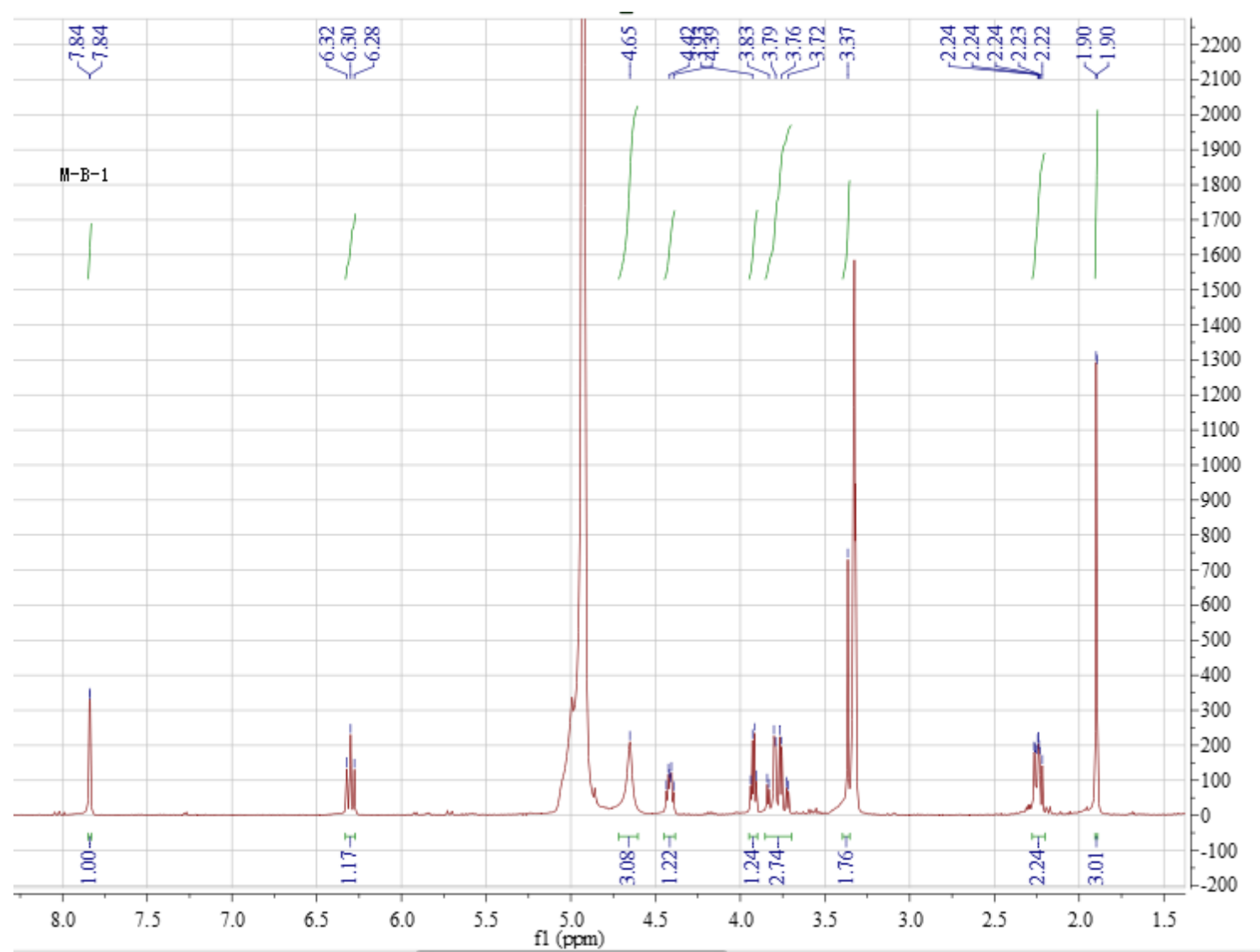

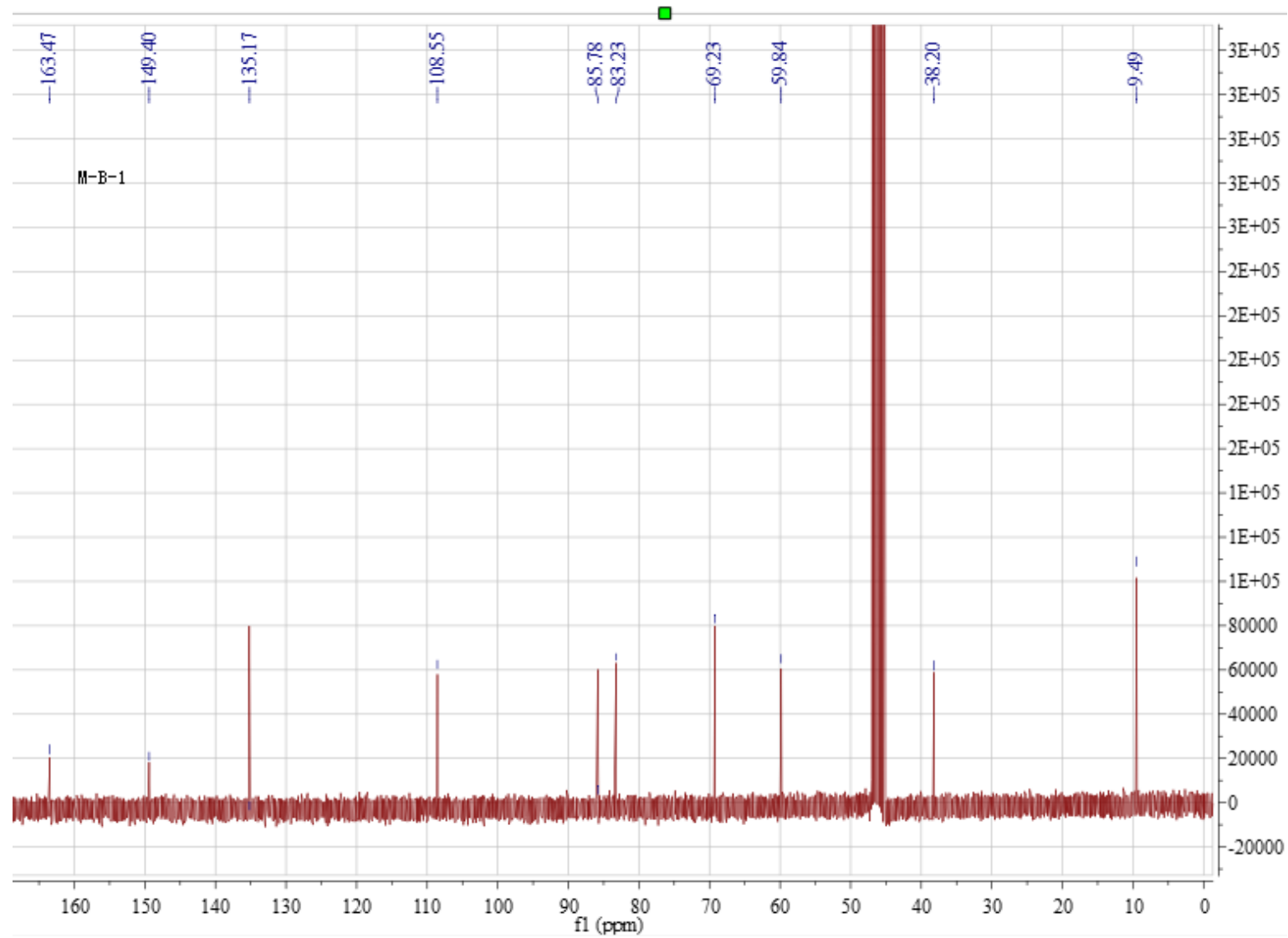

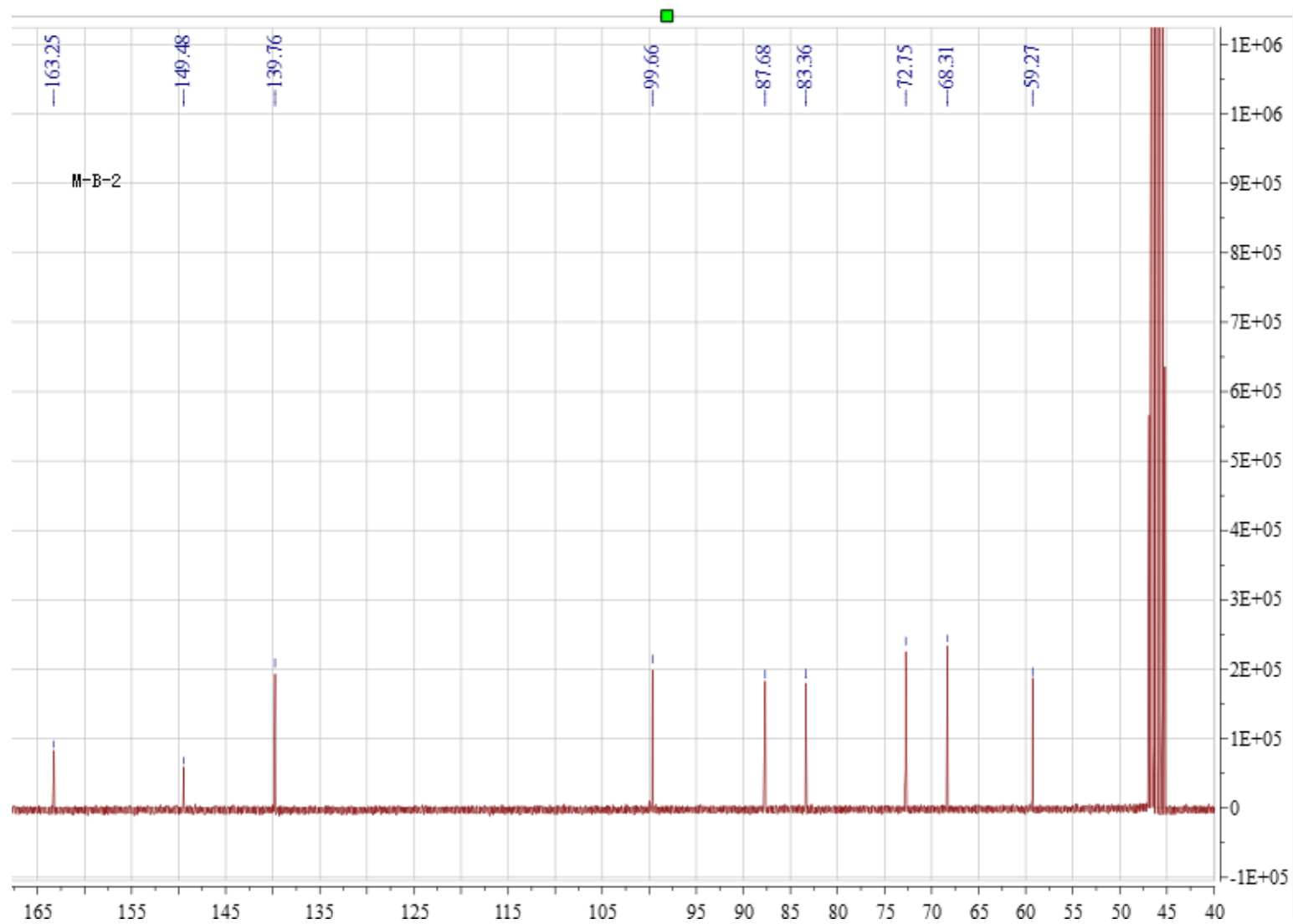

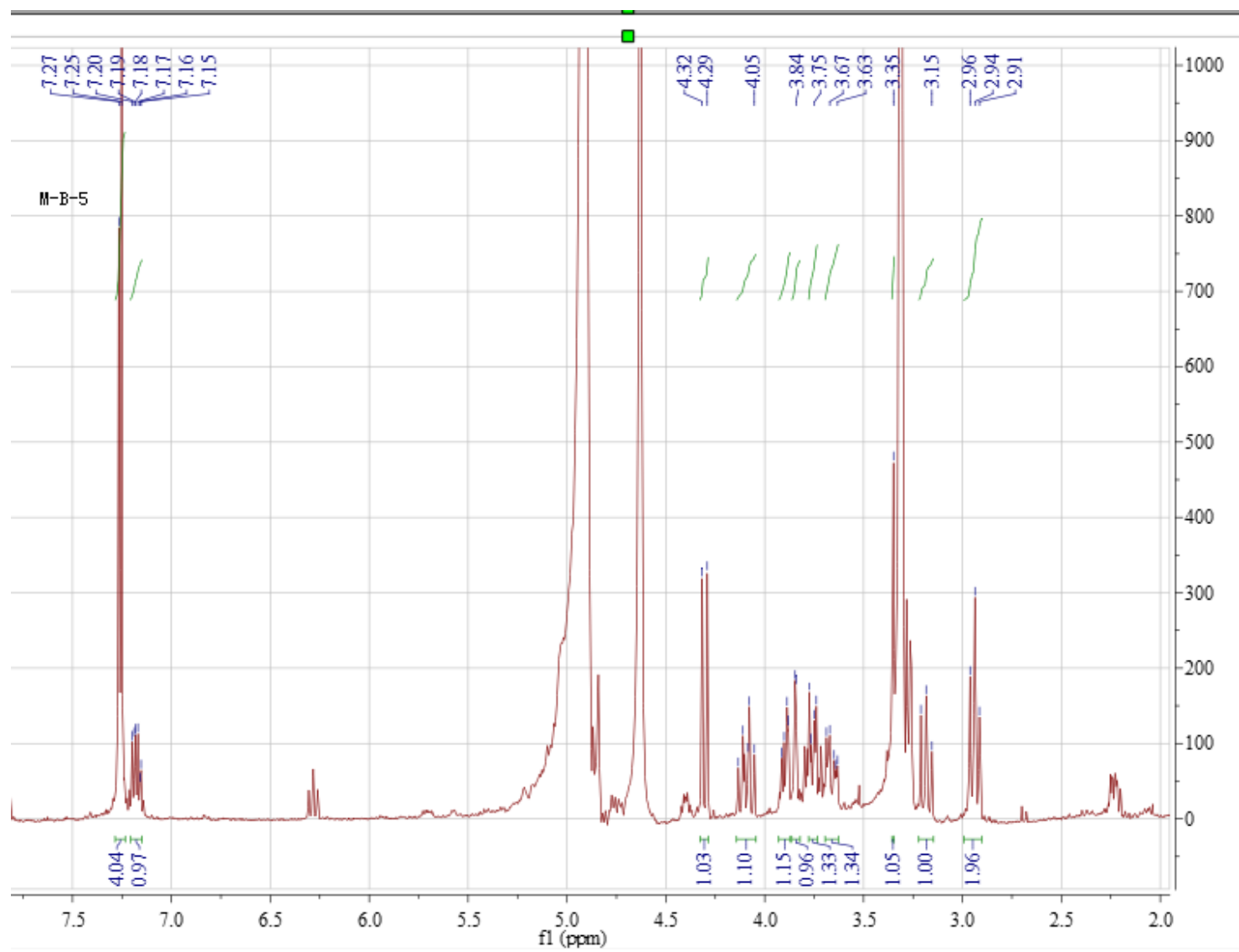

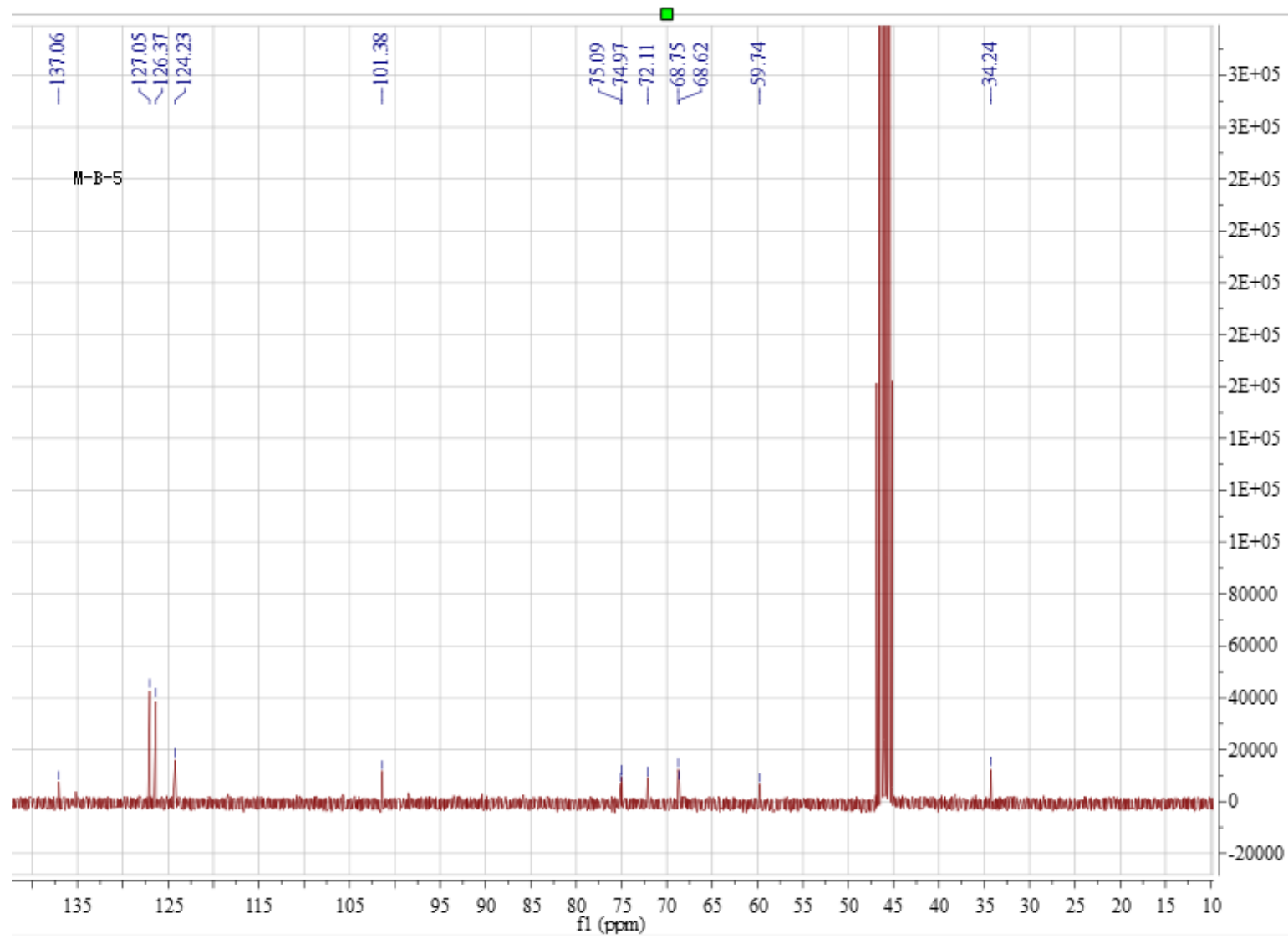

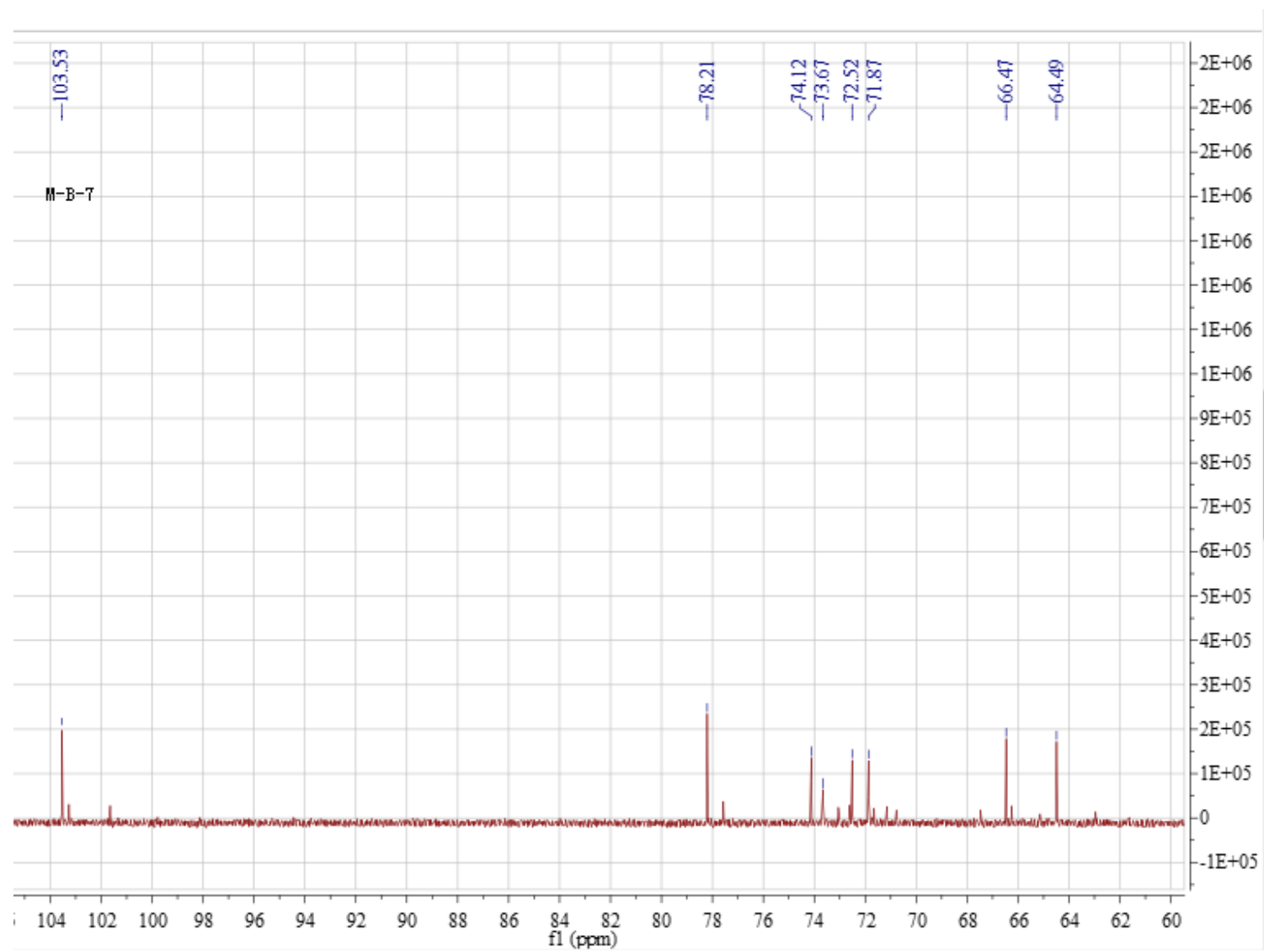

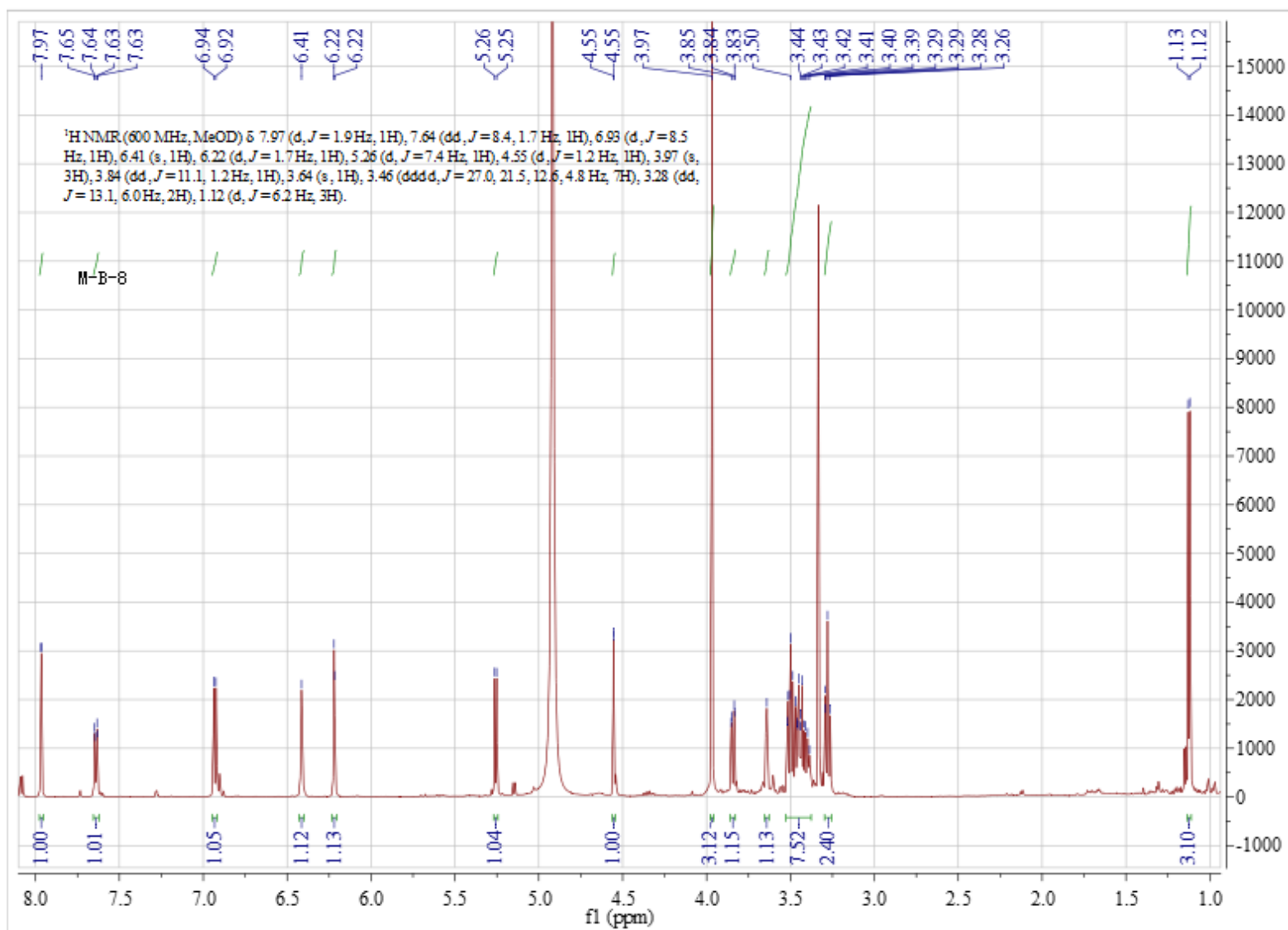

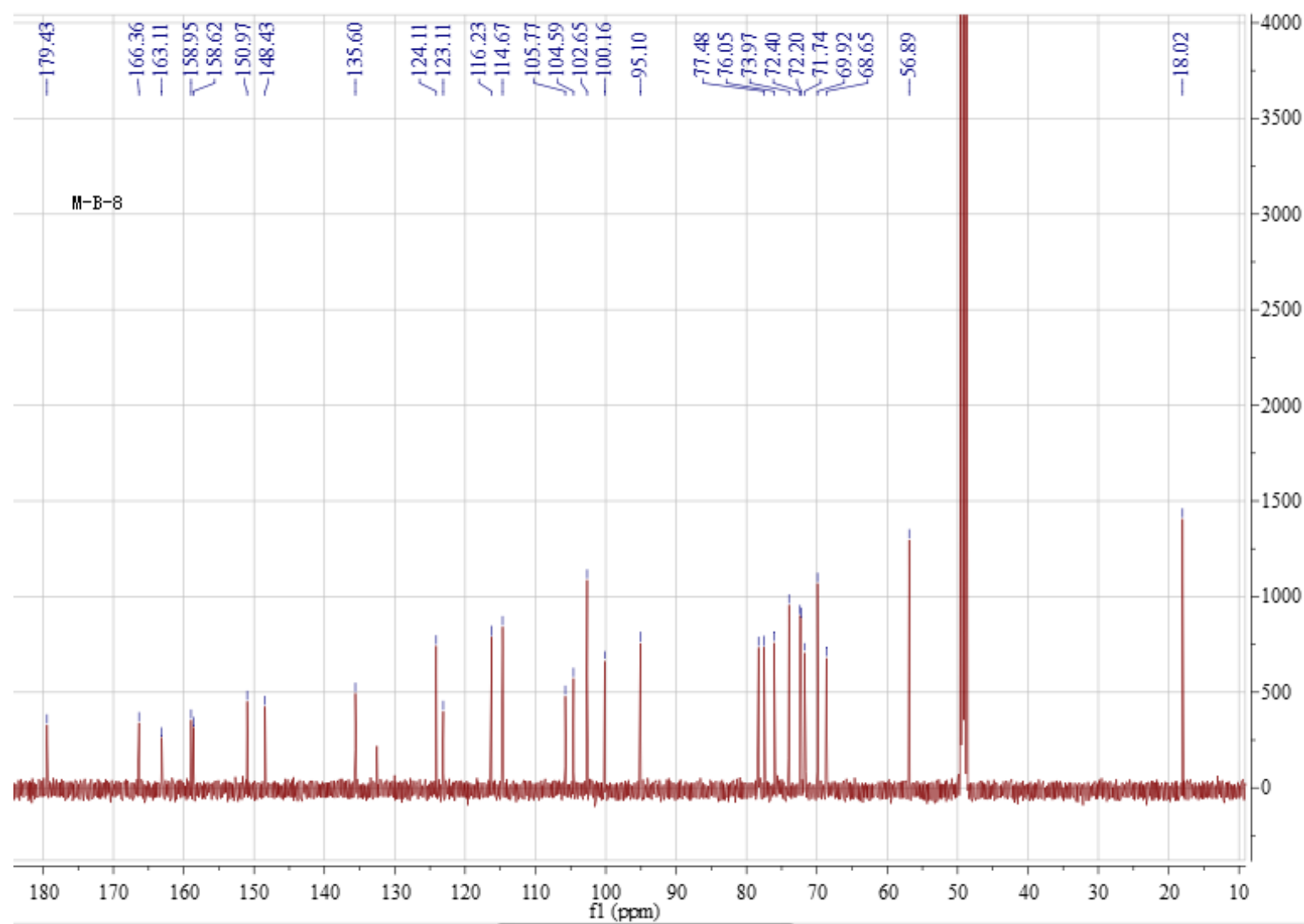

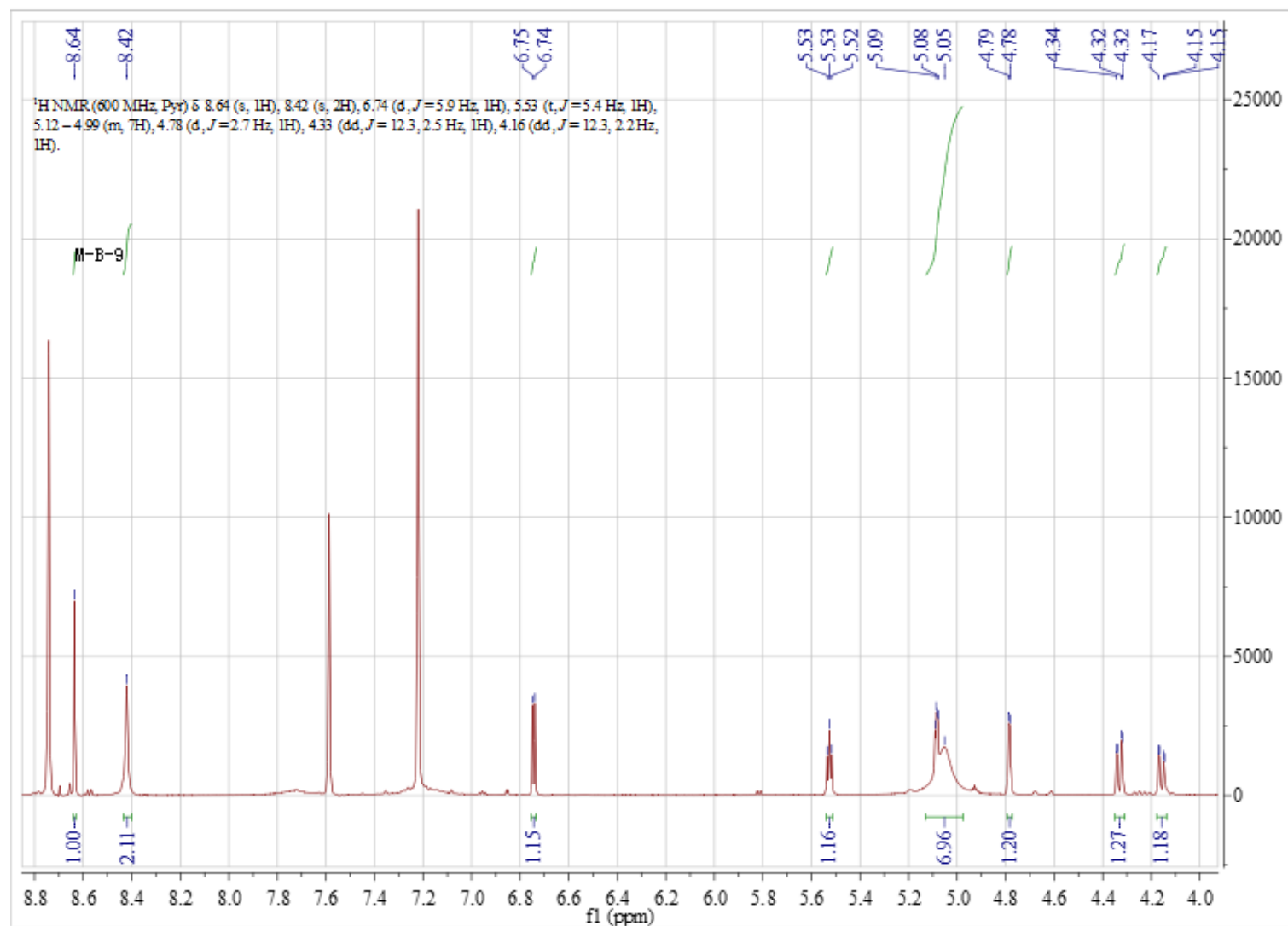

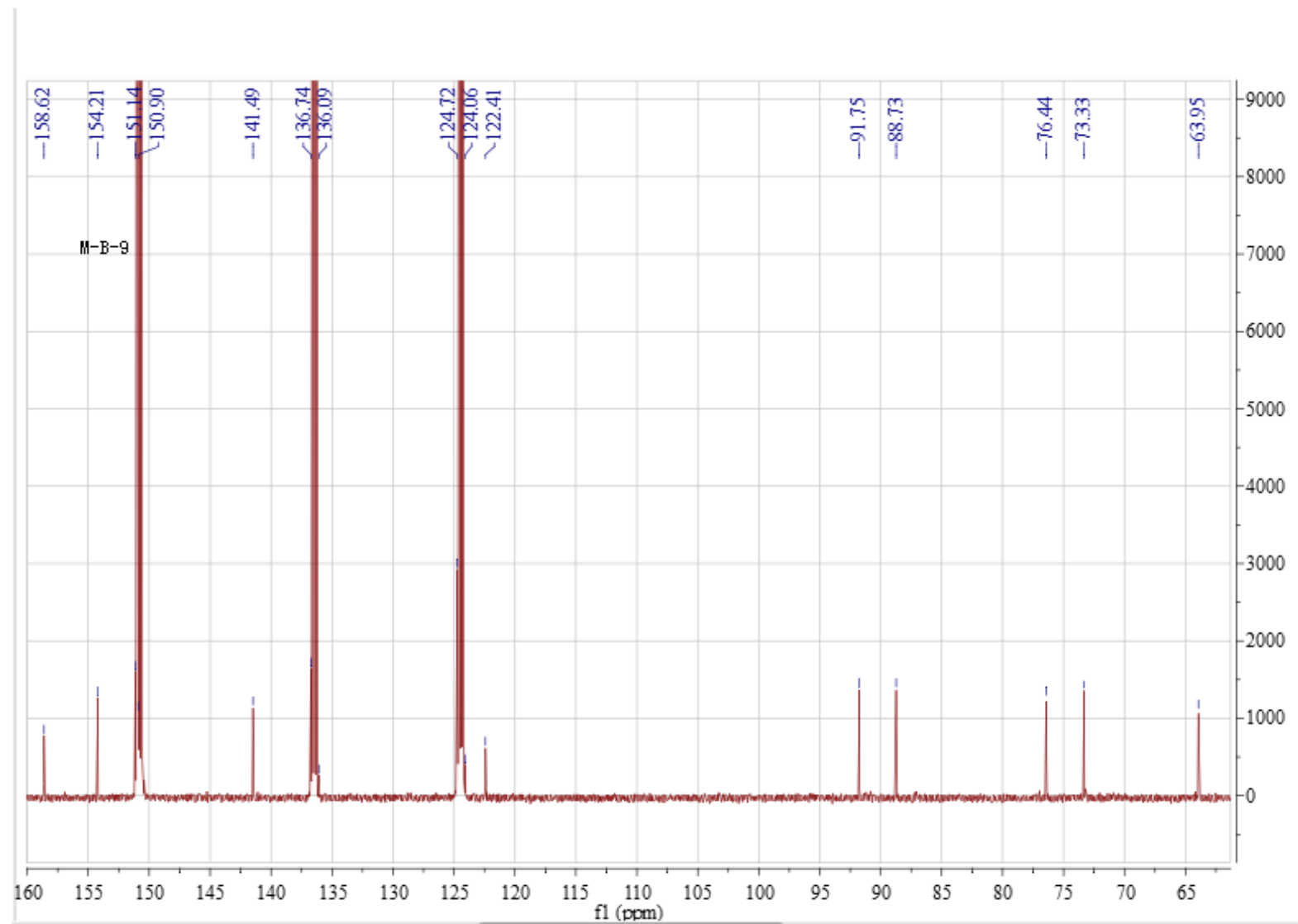

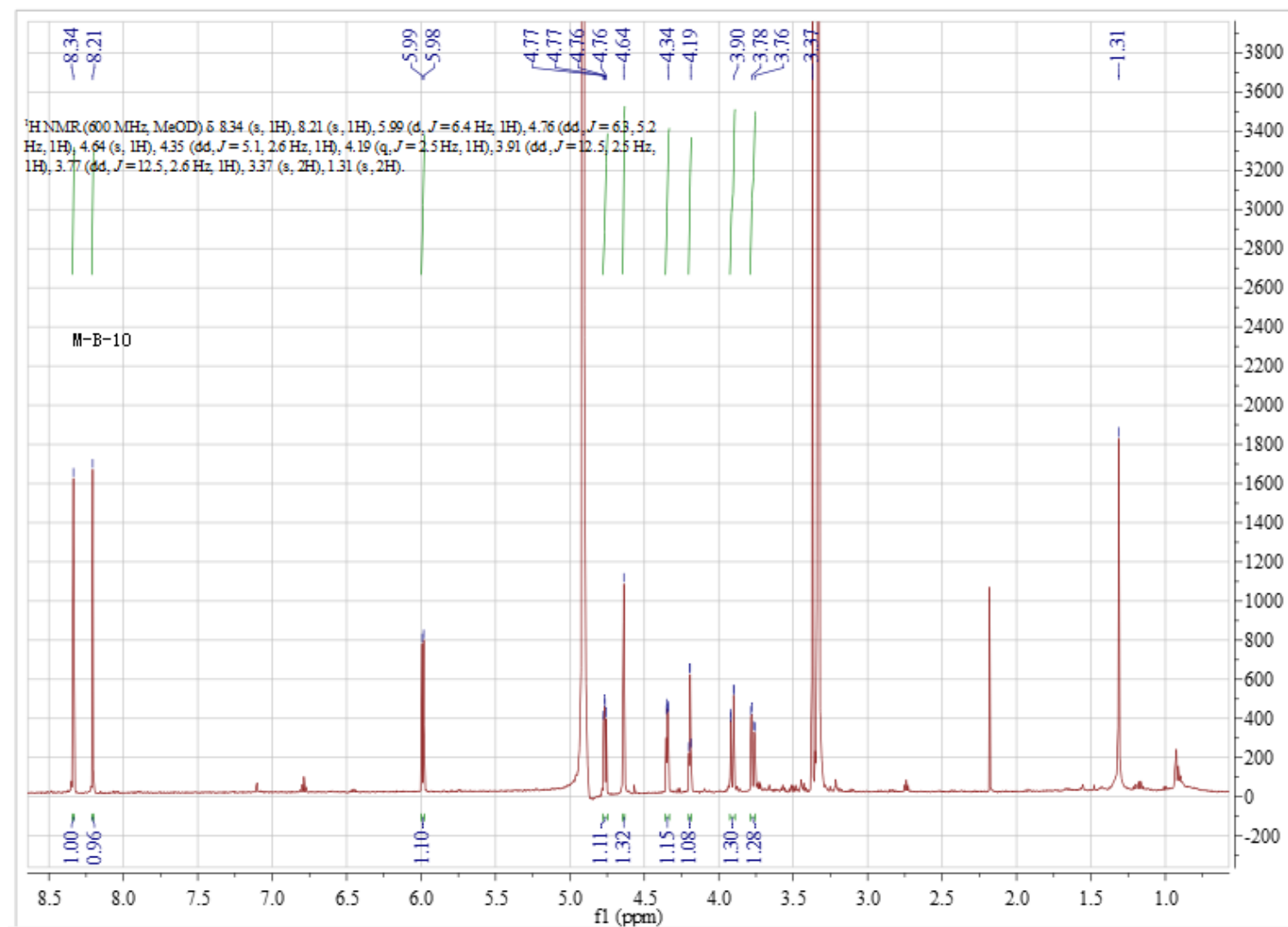

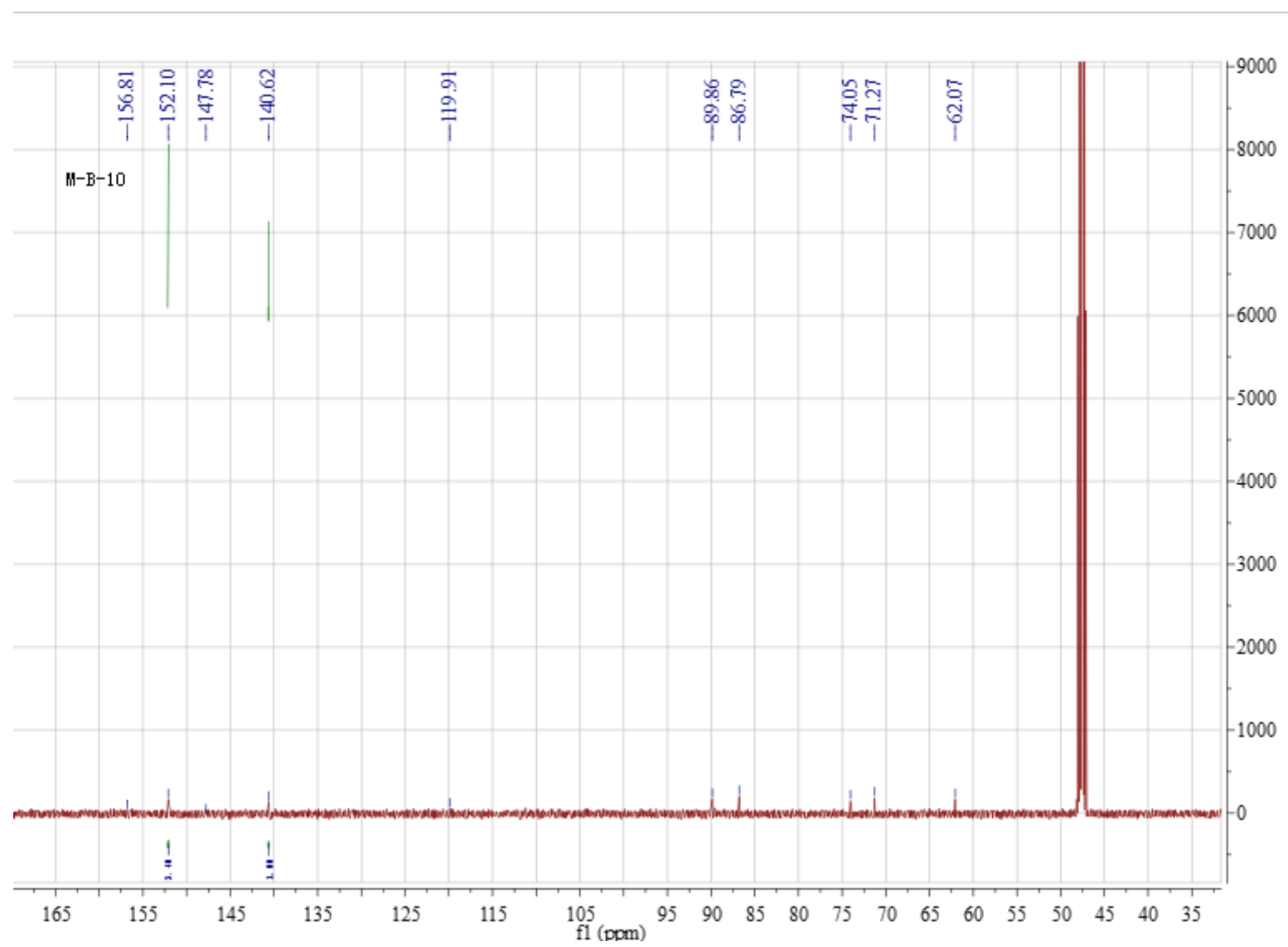

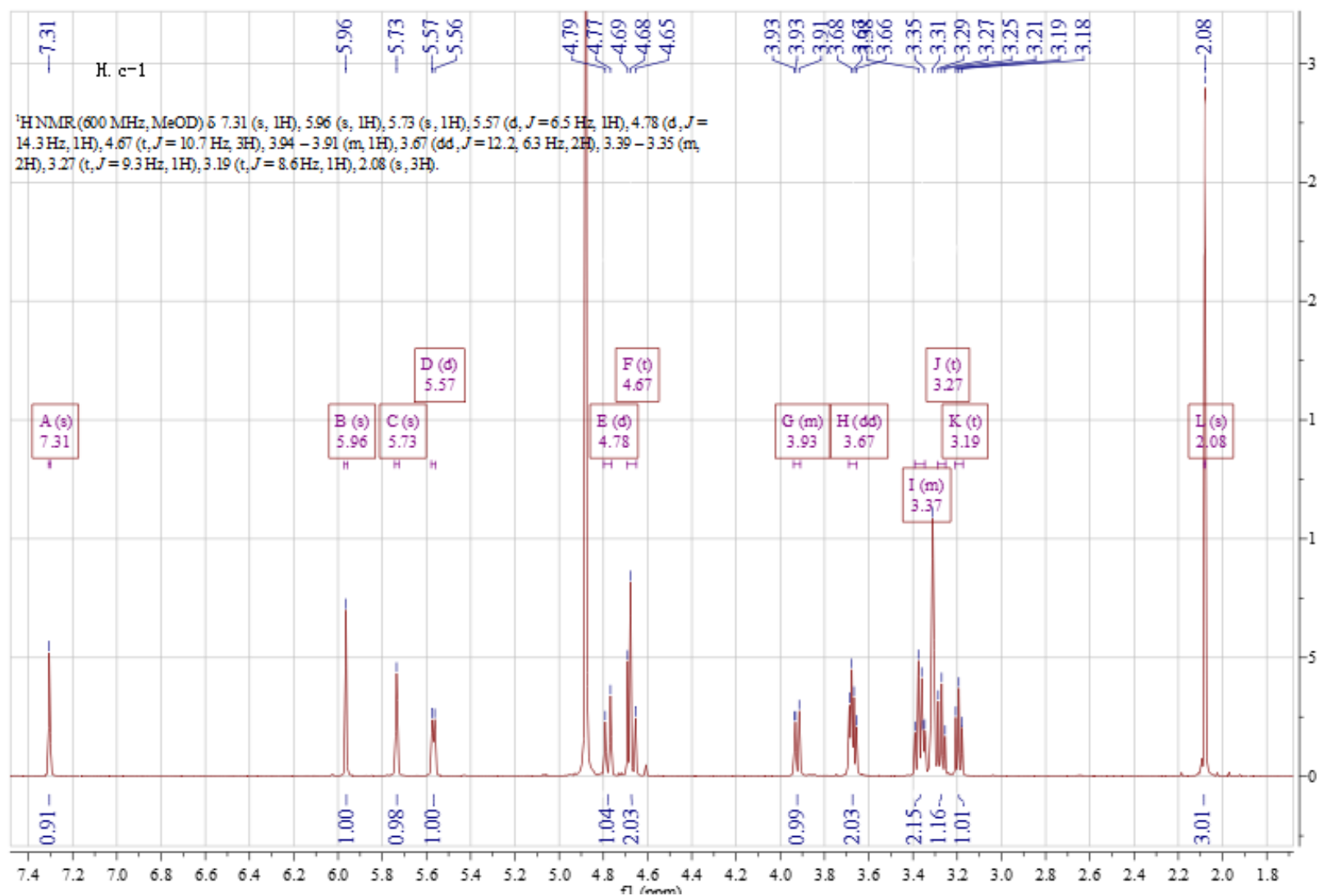

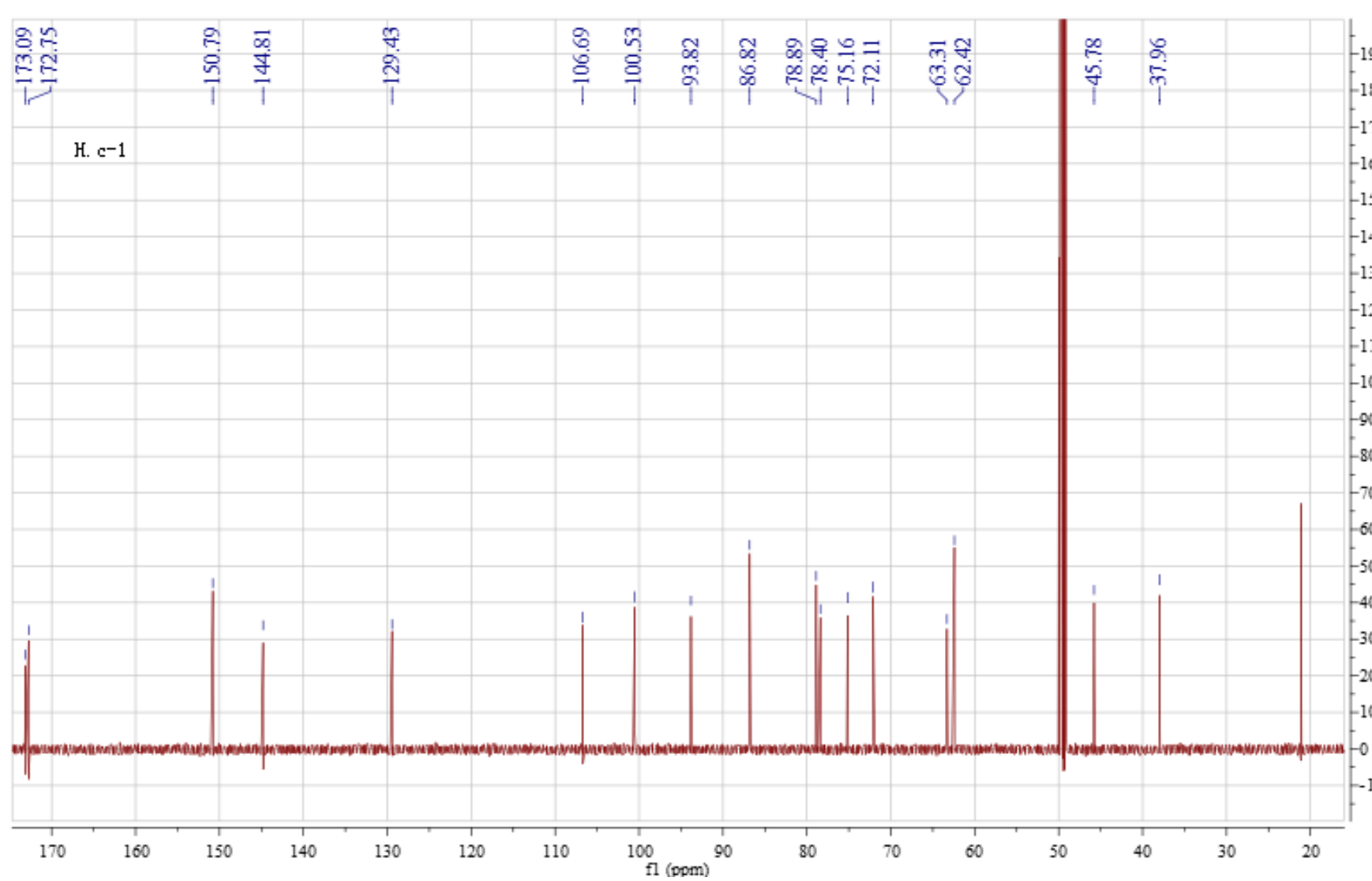

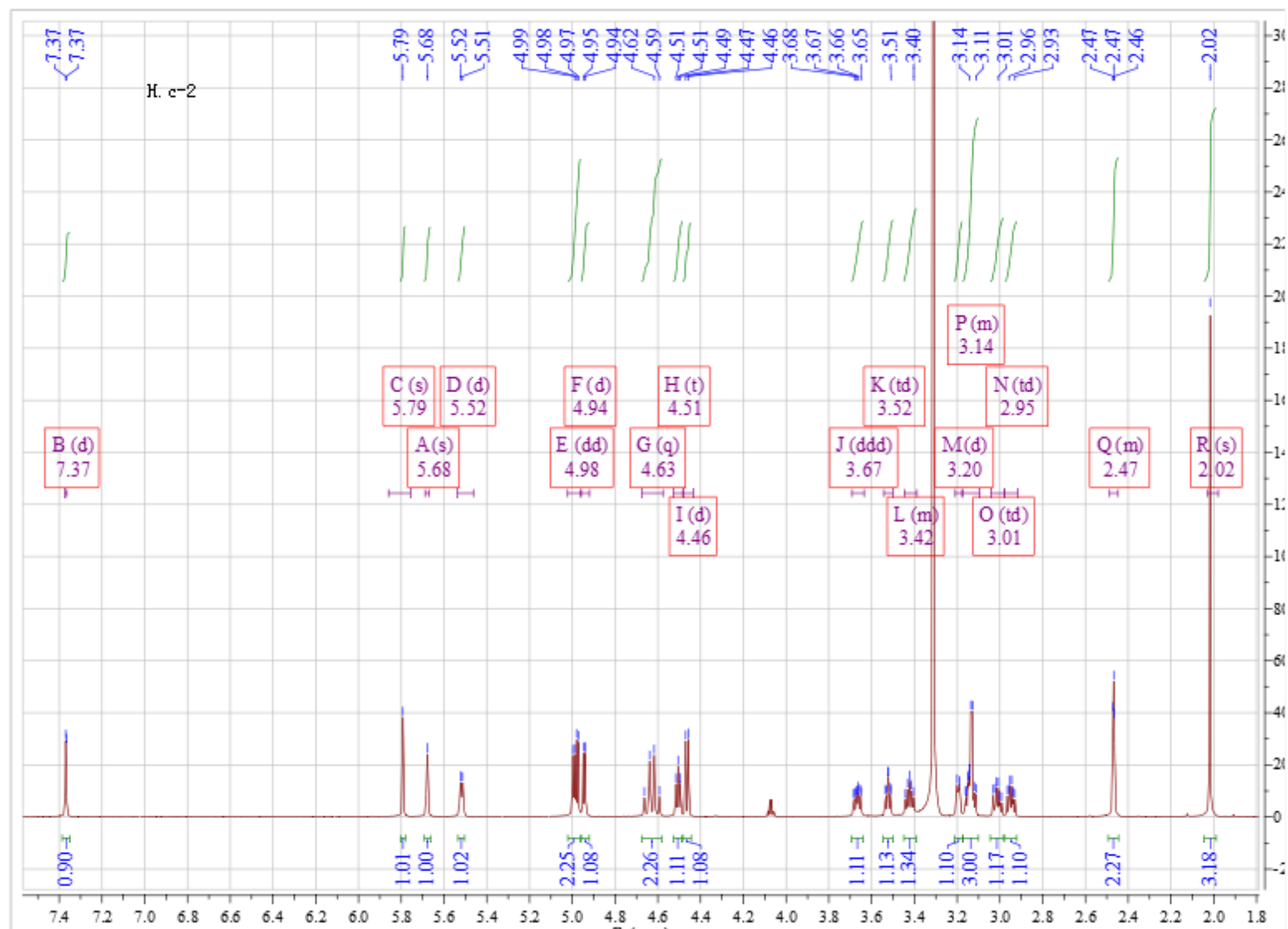

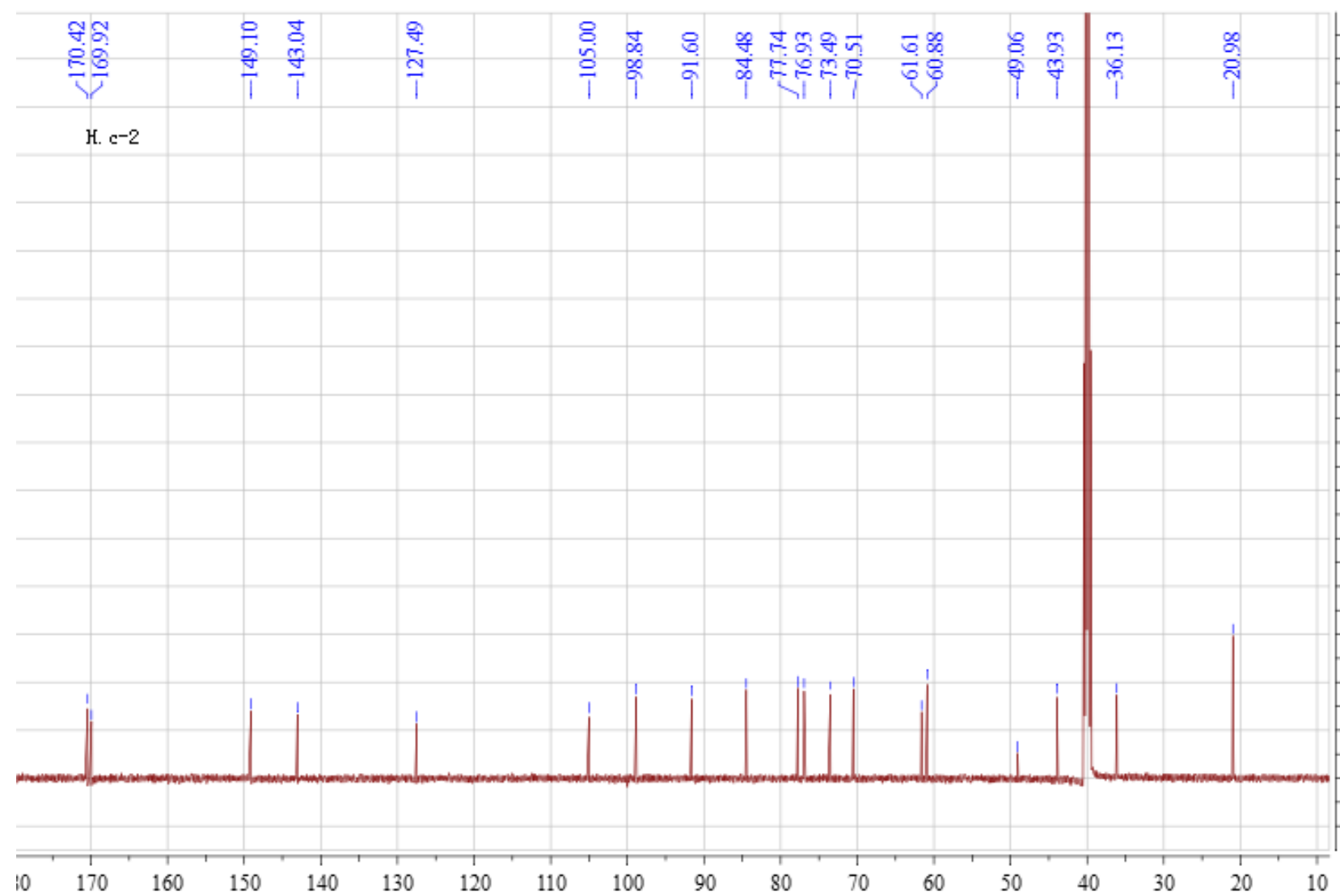

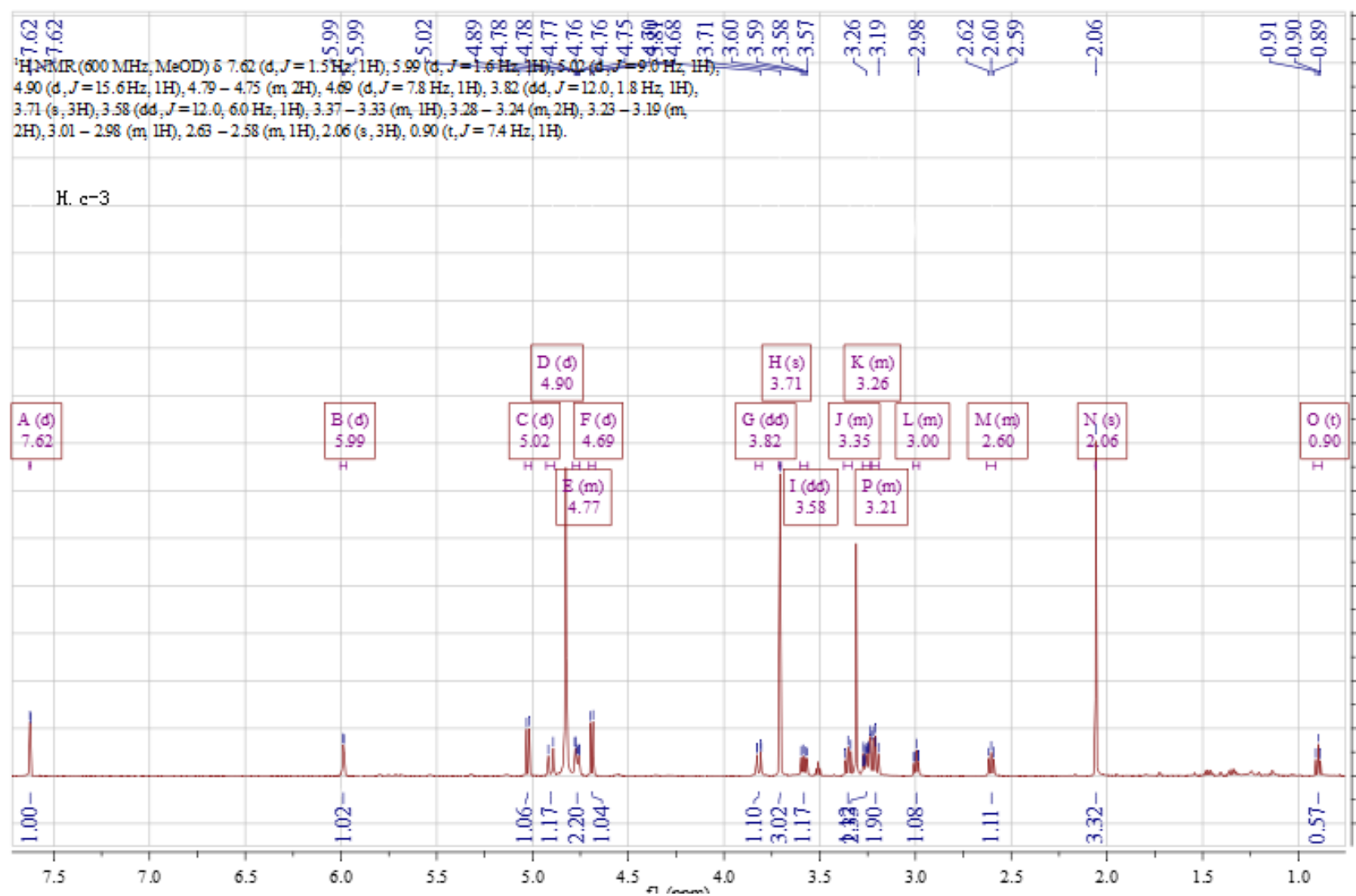

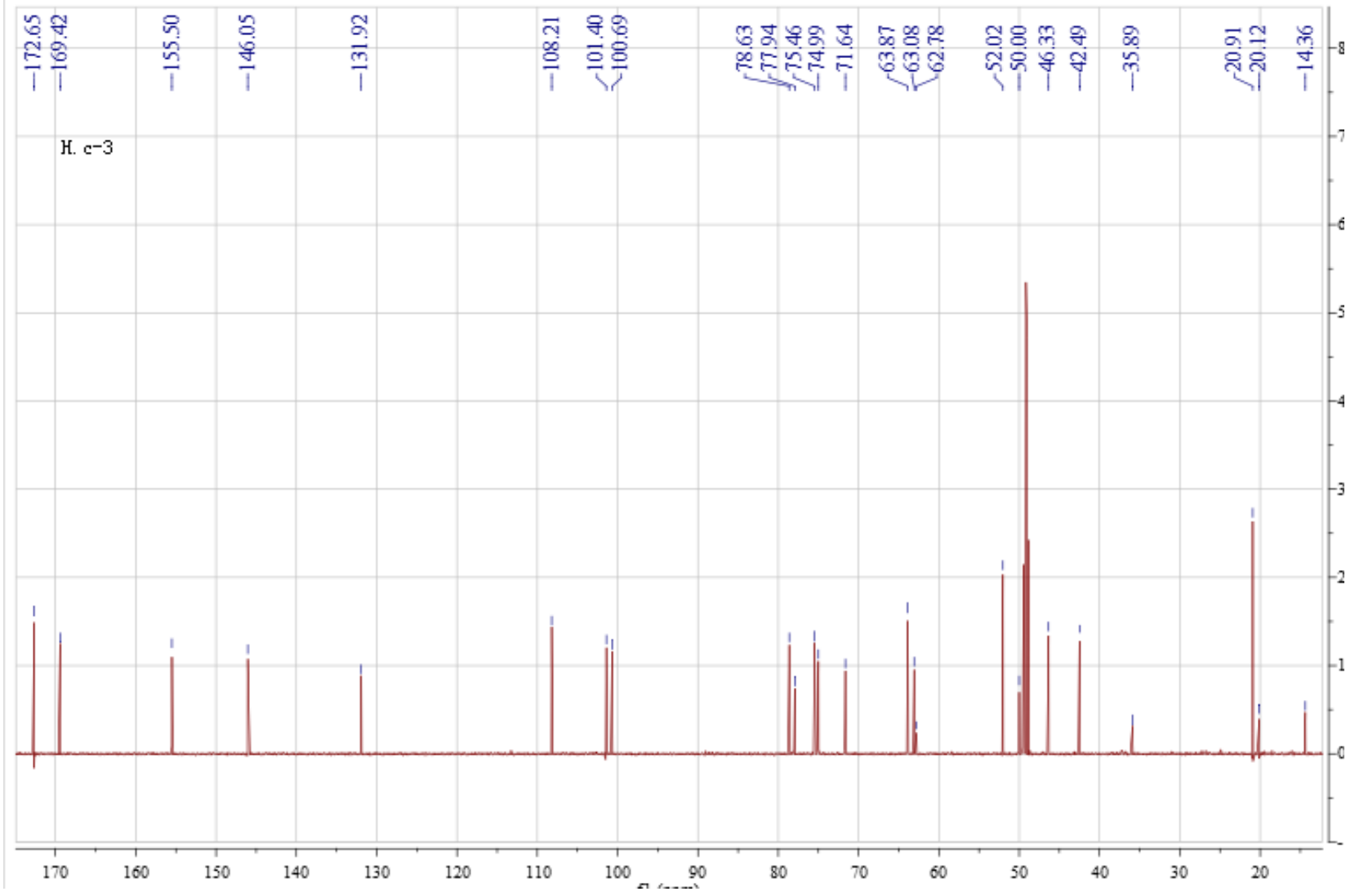

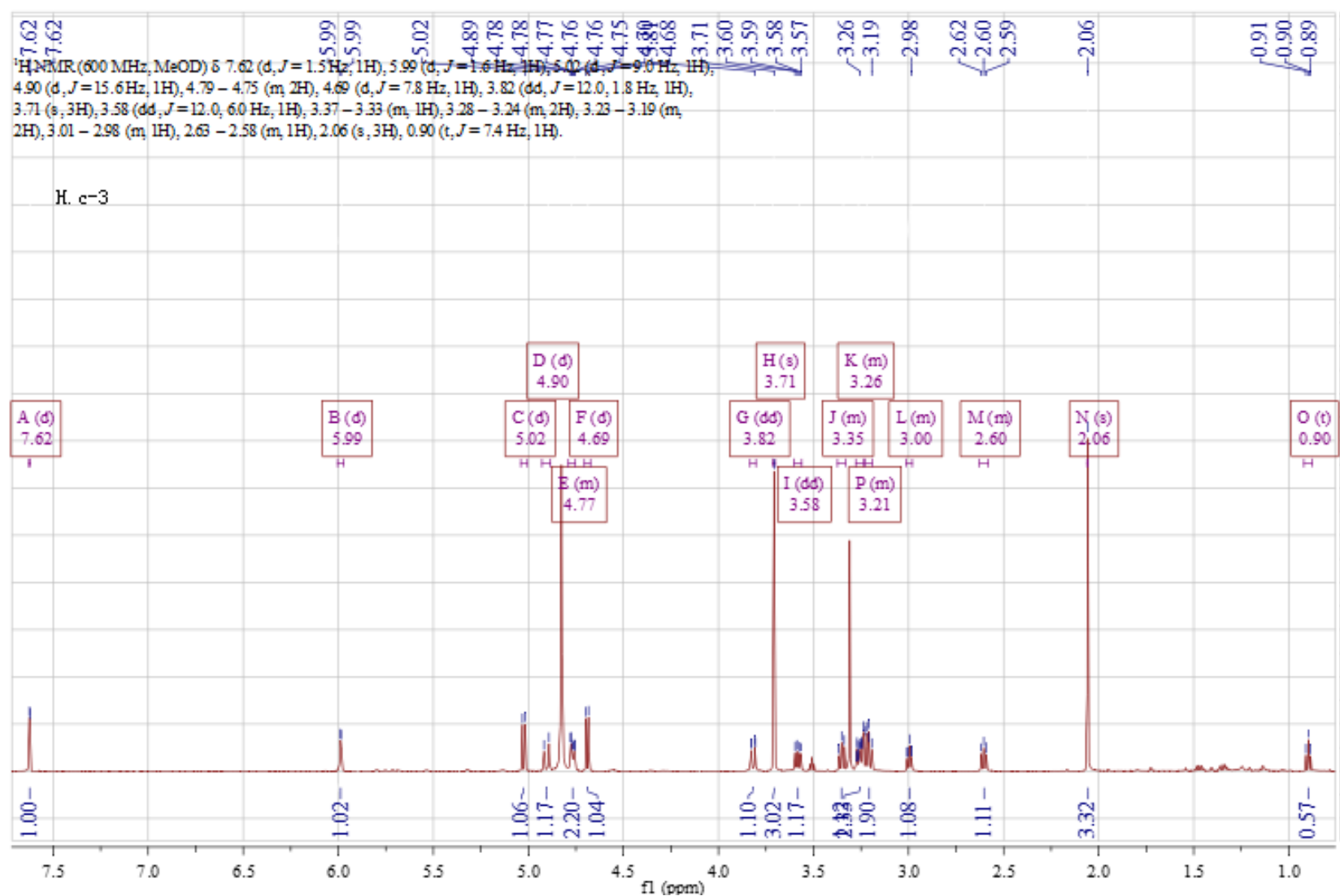

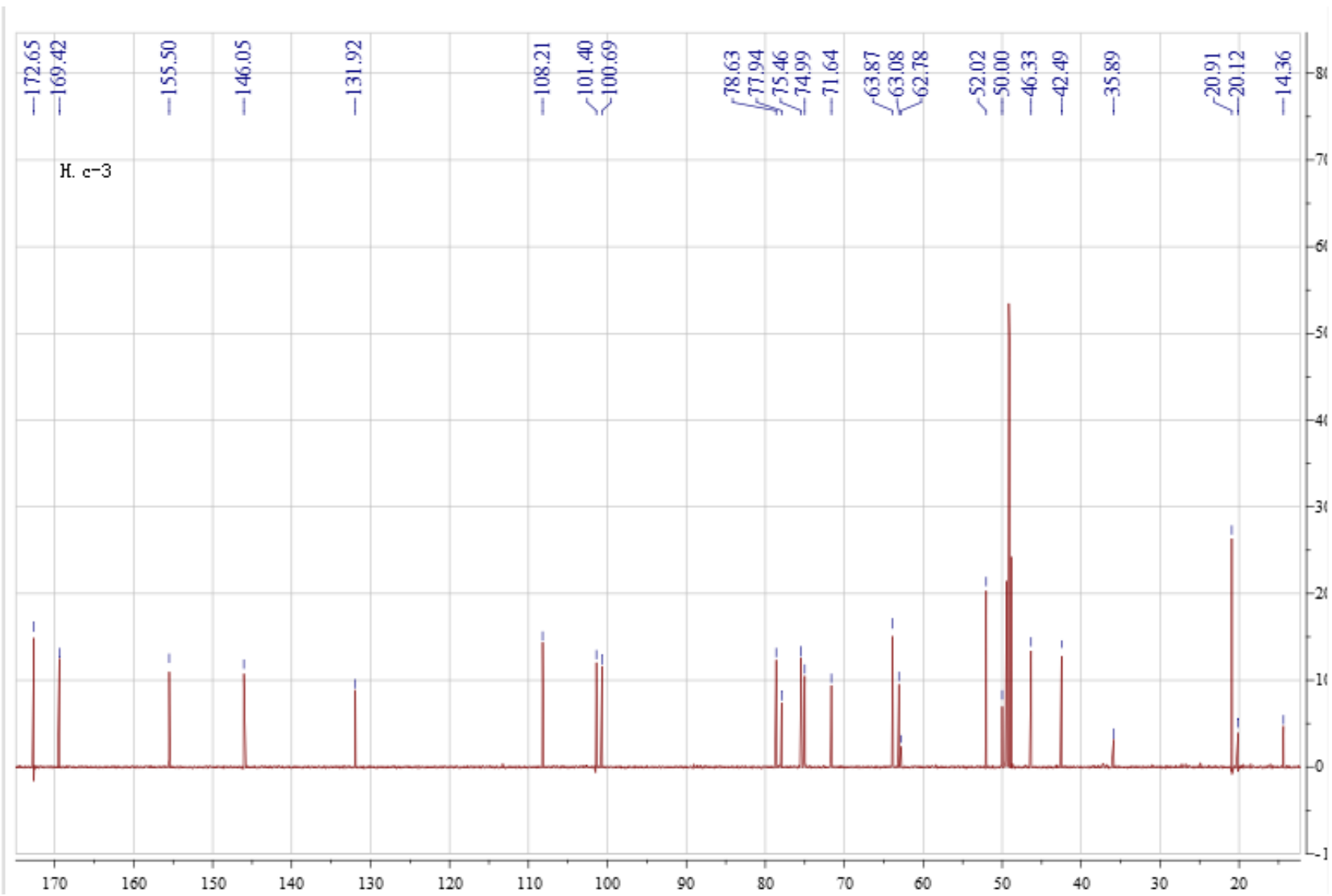

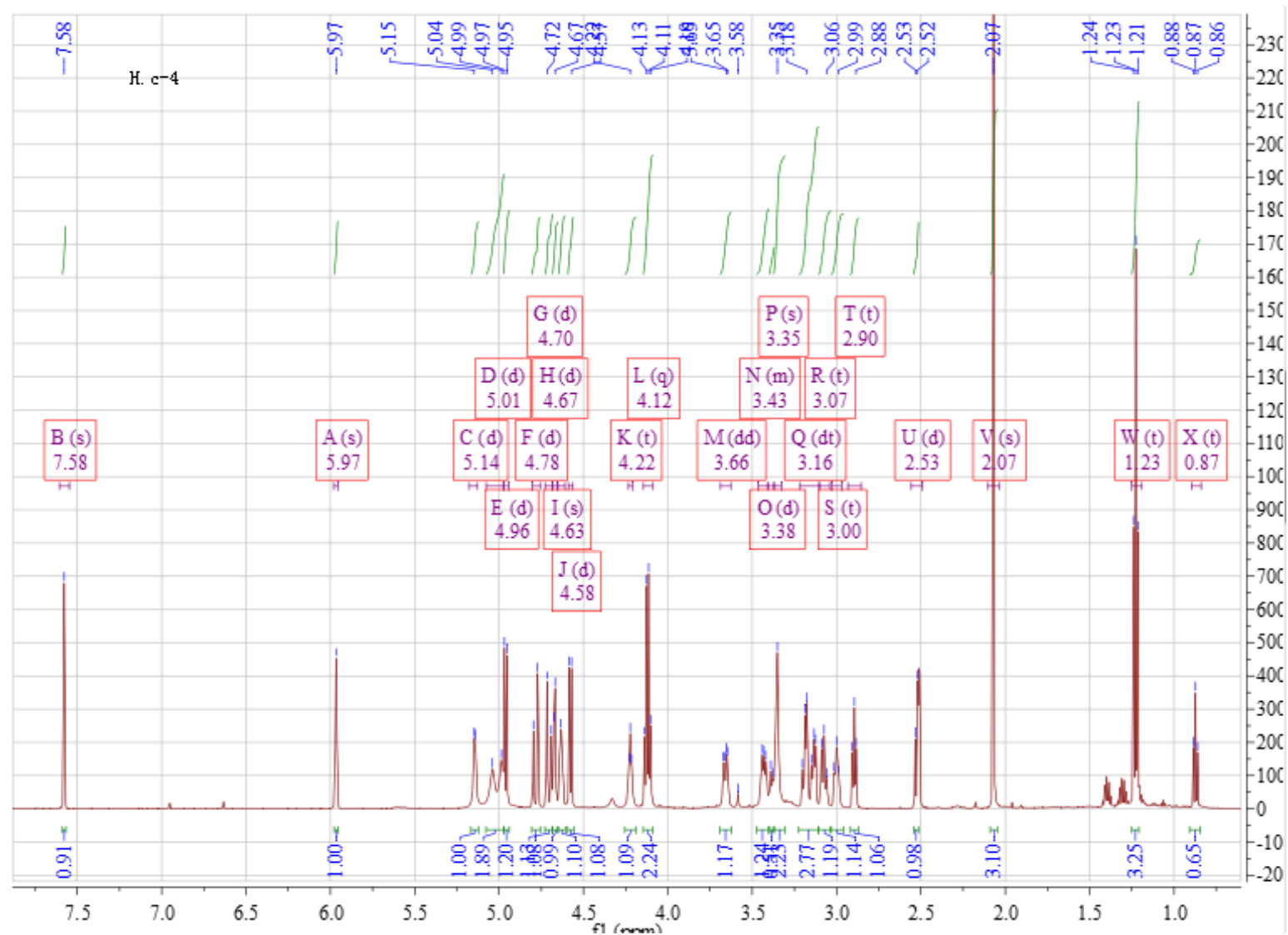

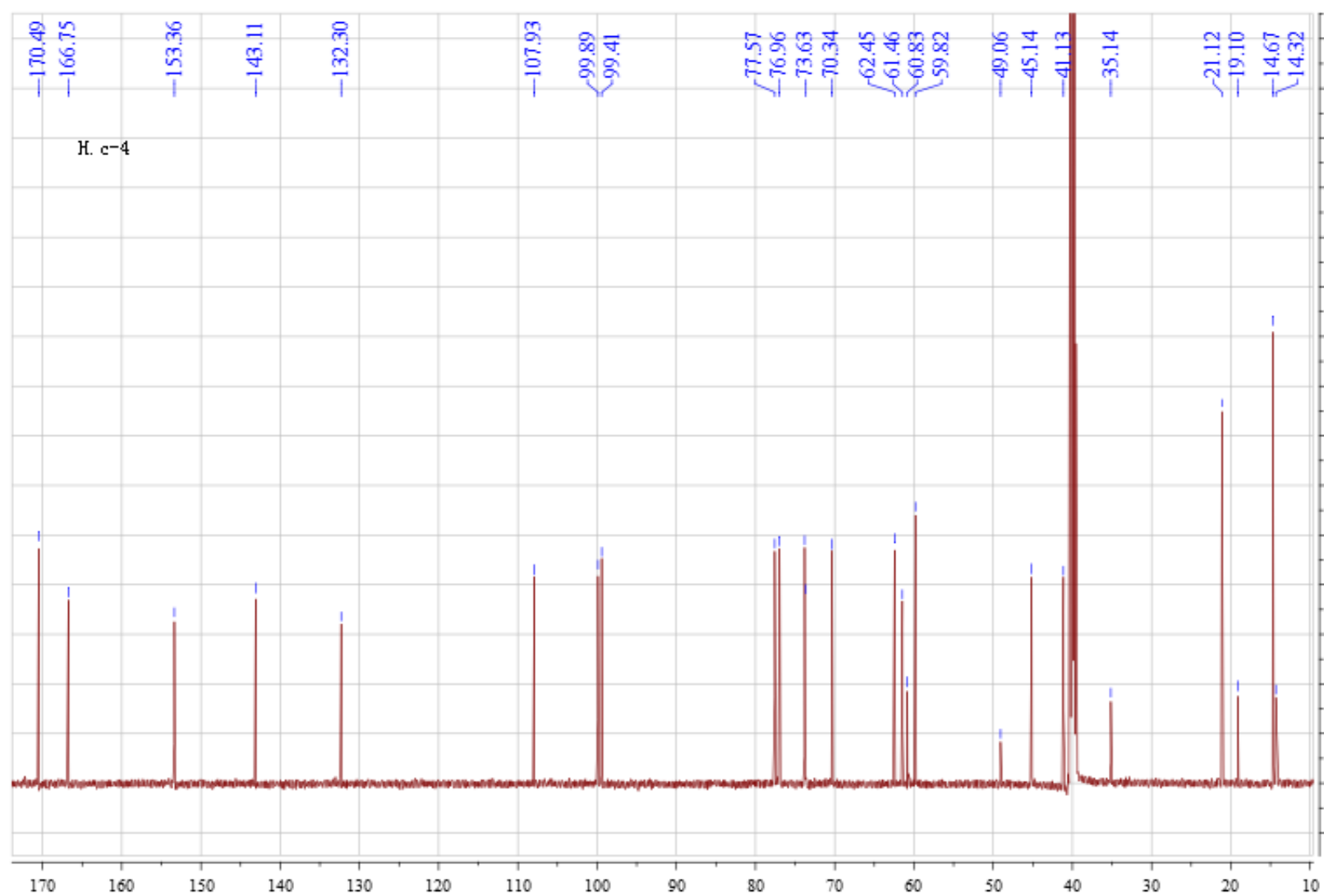

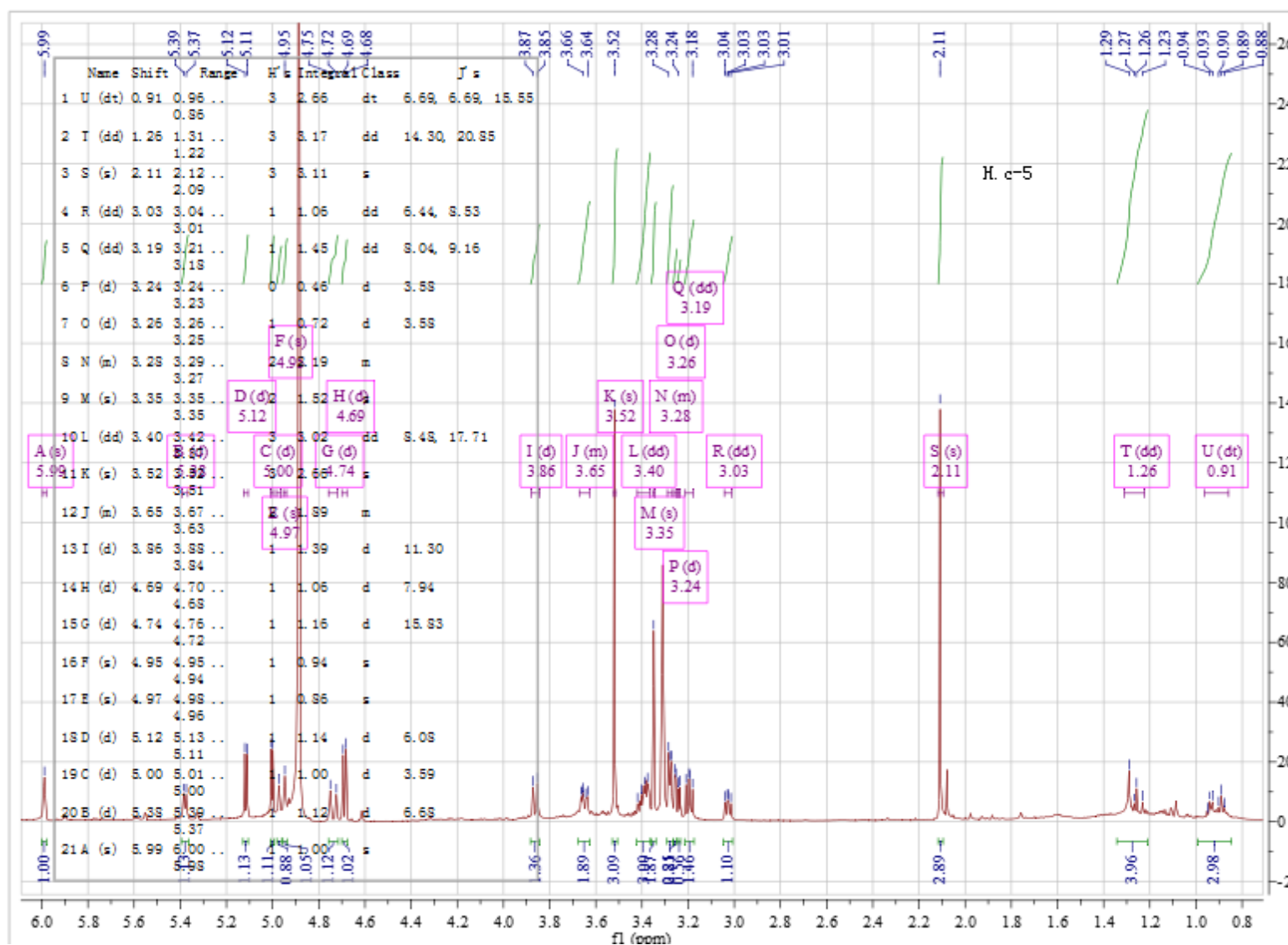

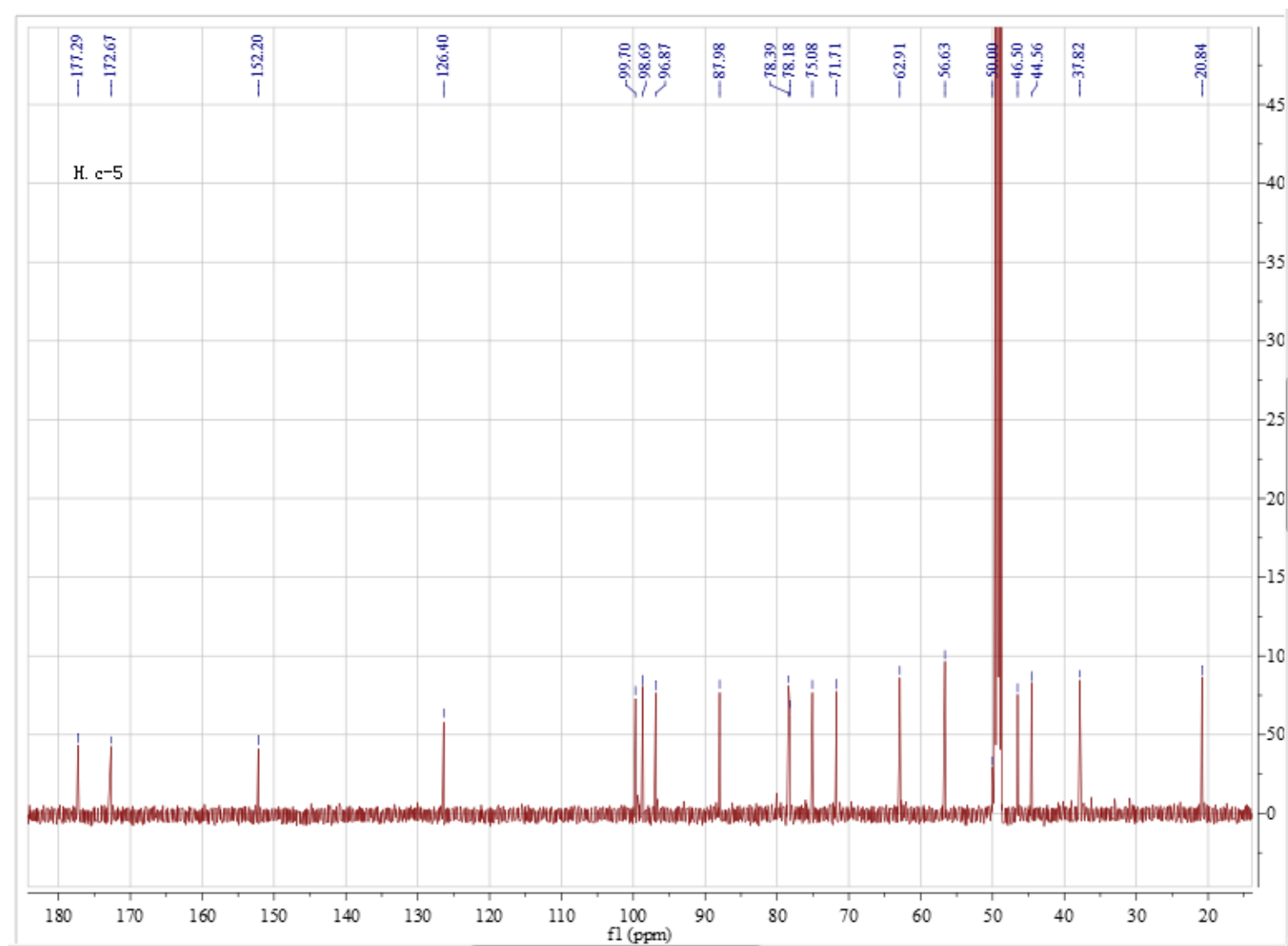

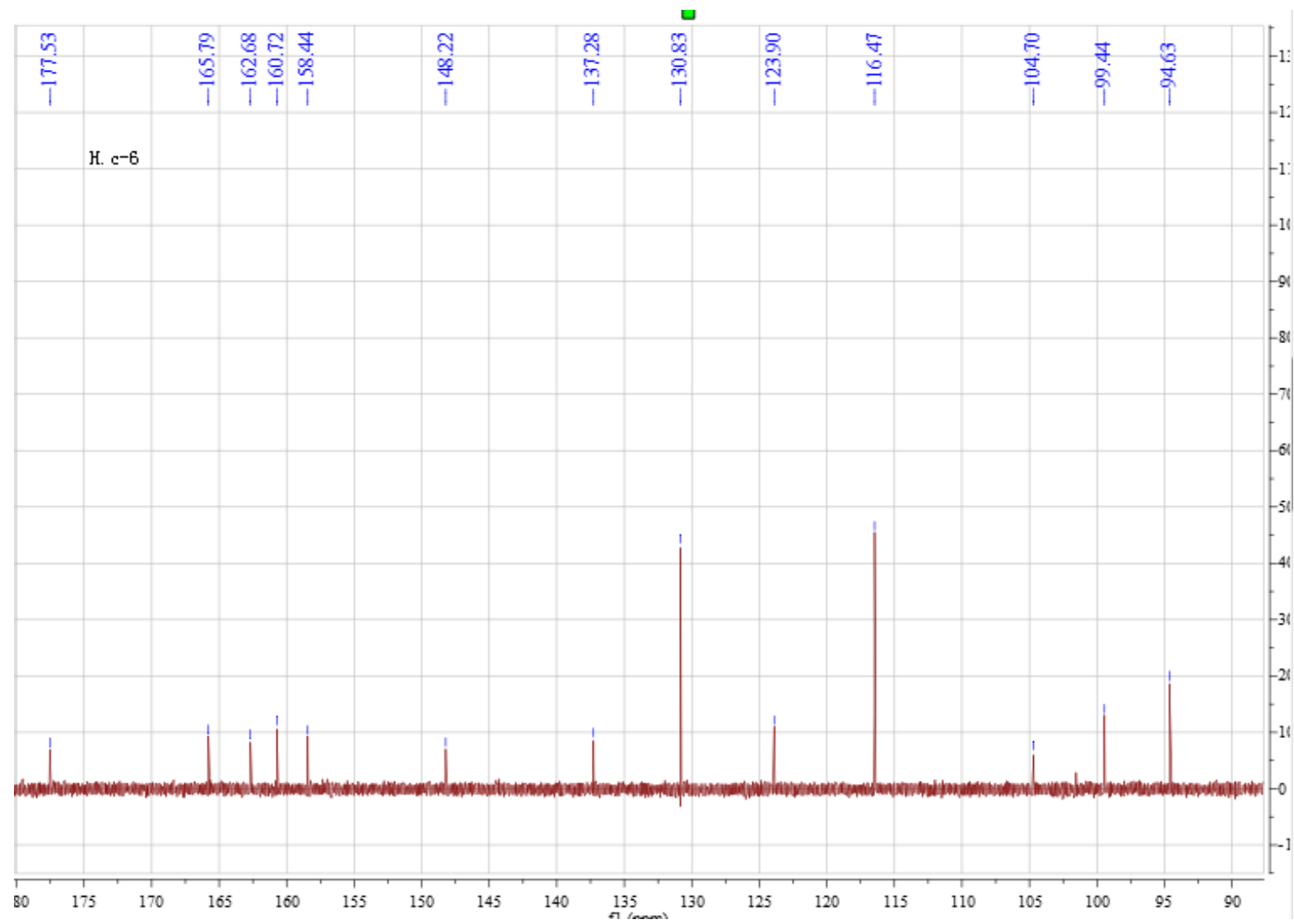

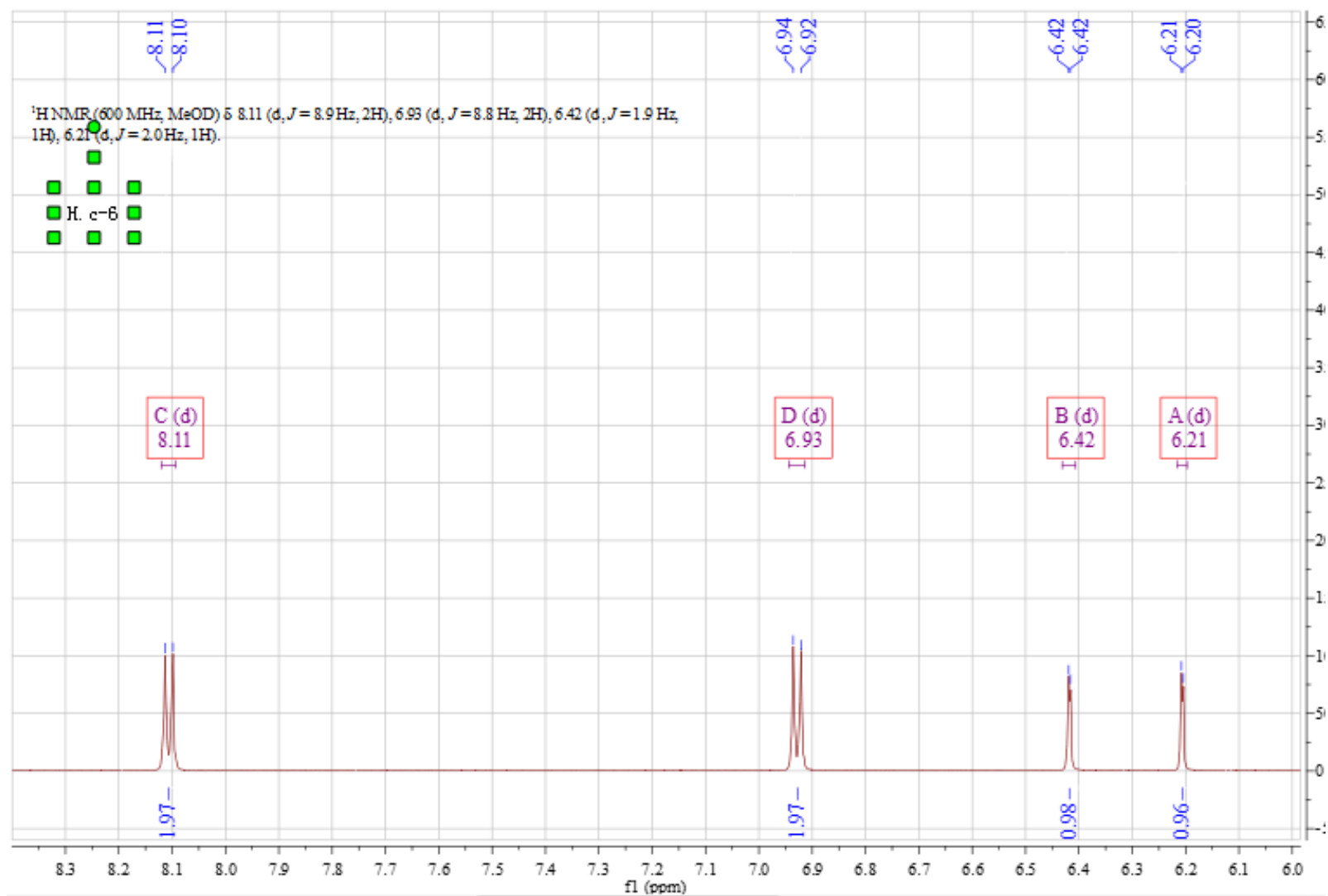

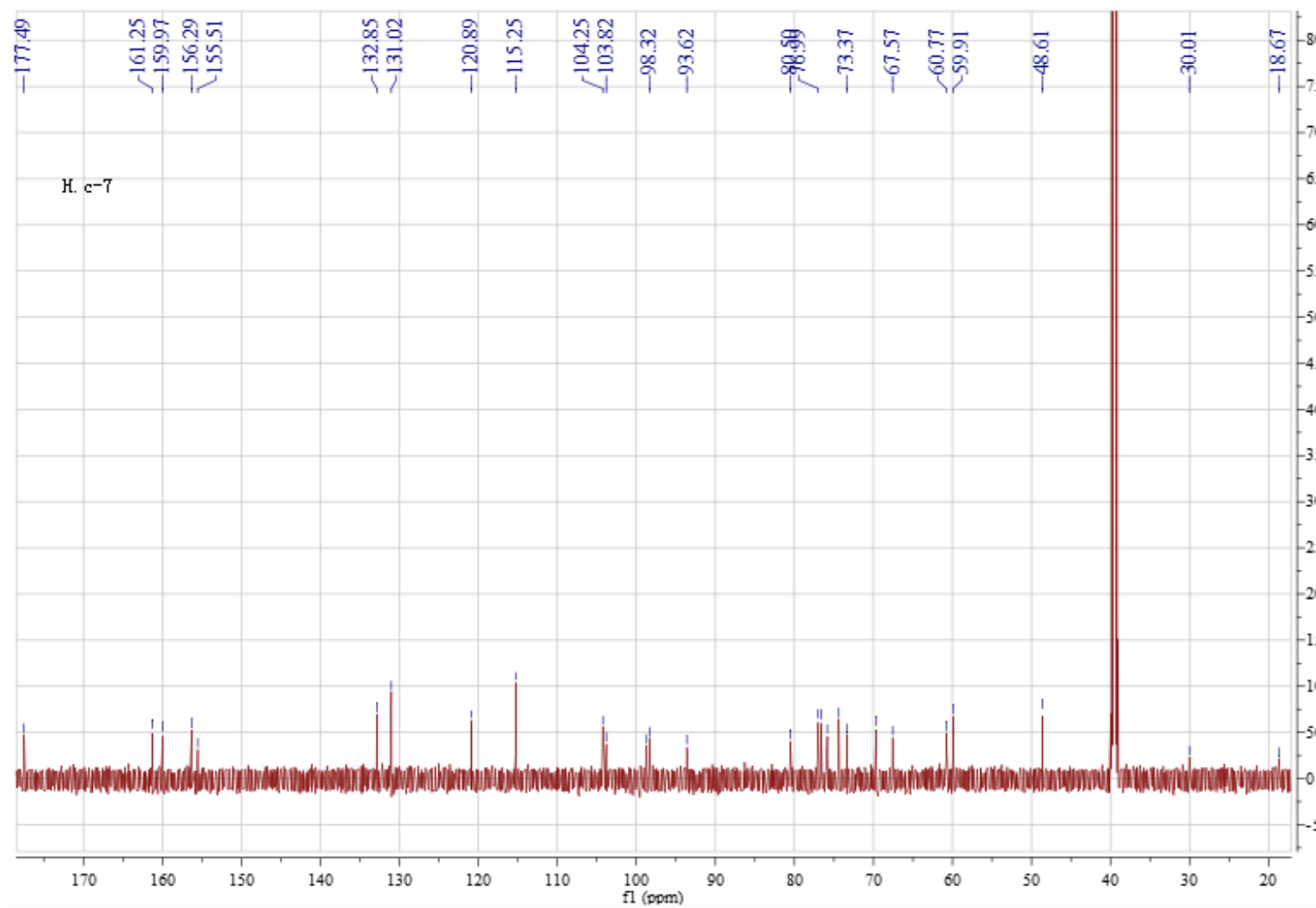

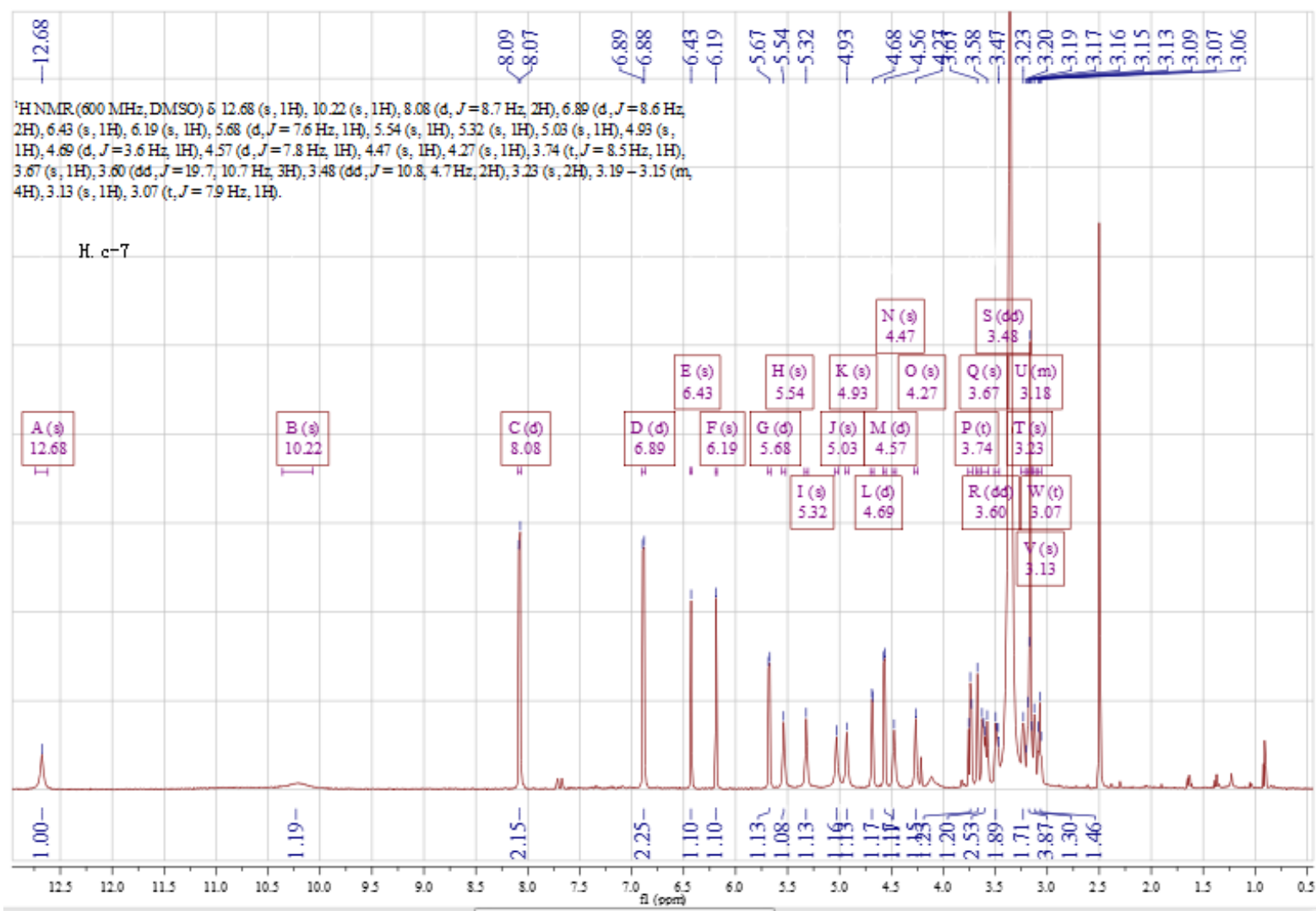

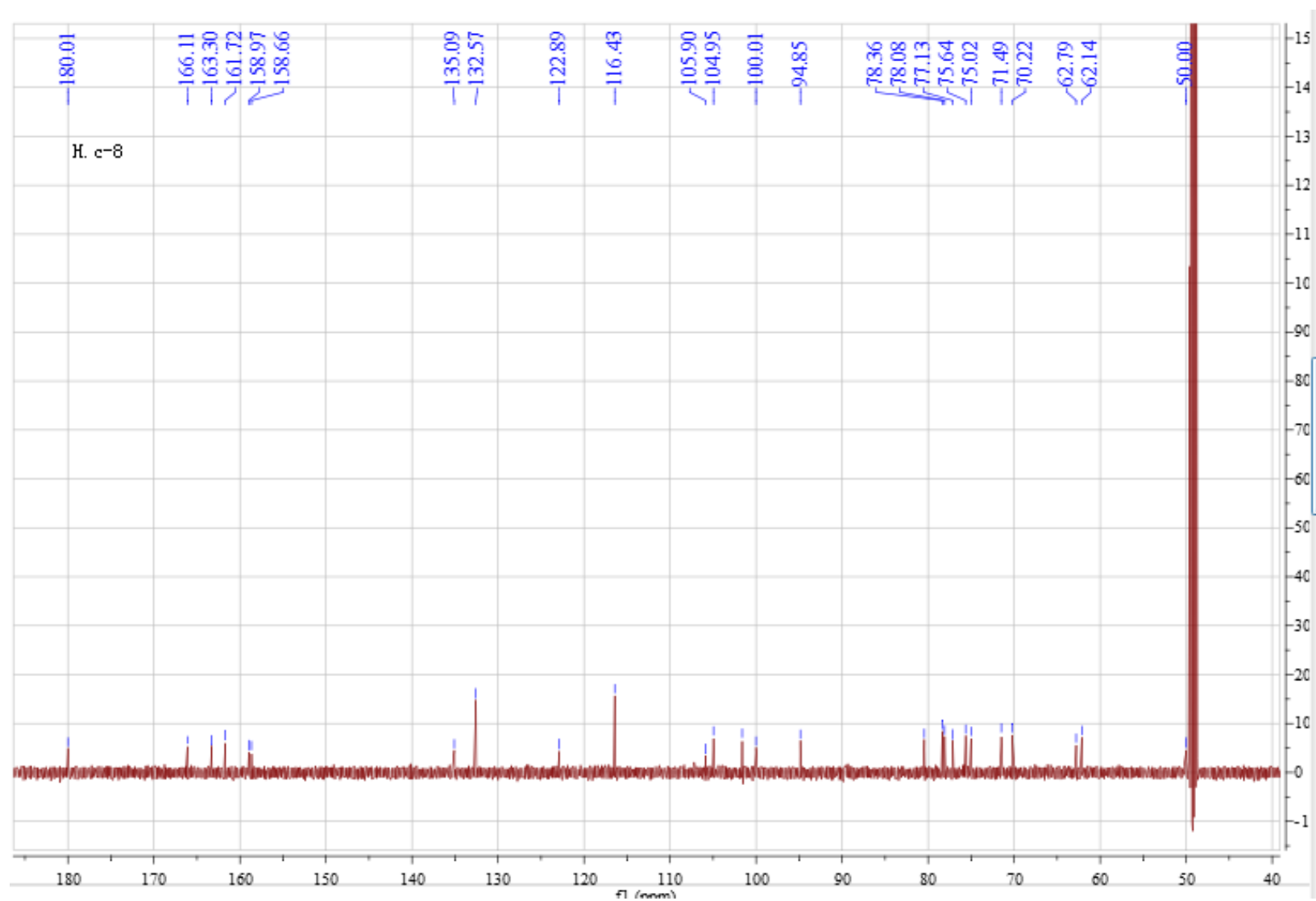

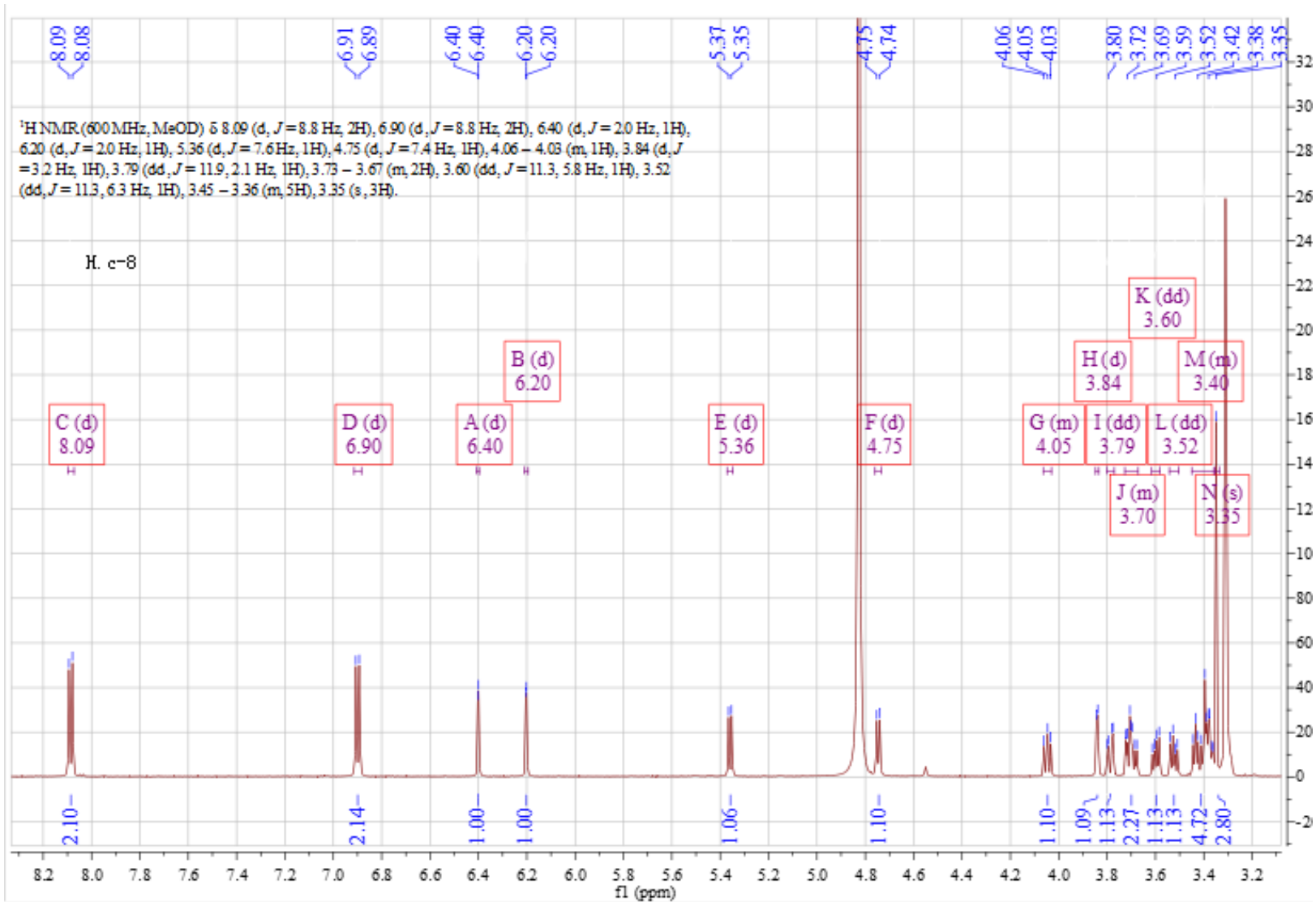

Supplement: Supplementary file 1 [file DataSheet1.PDF]
